# Pulse-Charging Energy Storage for Triboelectric Nanogenerator Based on Frequency Modulation

**DOI:** 10.1007/s40820-025-01714-3

**Published:** 2025-04-10

**Authors:** Kwon-Hyung Lee, Min-Gyun Kim, Woosuk Kang, Hyun-Moon Park, Youngmin Cho, Jeongsoo Hong, Tae-Hee Kim, Seung-Hyeok Kim, Seok-Kyu Cho, Donghyeon Kang, Sang-Woo Kim, Changshin Jo, Sang-Young Lee

**Affiliations:** 1 Department of Chemical and Biomolecular Engineering, Yonsei University, Seoul, 03722 Republic of Korea; 2 Ulsan Advanced Energy Technology R&D Center, Korea Institute of Energy Research (KIER), Ulsan, 44776 Republic of Korea; 3 Department of Battery Engineering, Pohang University of Science and Technology (POSTECH), Pohang, 37673 Republic of Korea; 4 Research and Development Center, Energy-Mining LTD, Suwon, 16226 Republic of Korea; 5 Department of Chemical Engineering, POSTECH, Pohang, 37673 Republic of Korea; 6 Department of Battery and Chemical Engineering, Hanyang University ERICA, Gyeonggi, 15588 Republic of Korea; 7 UBATT Inc, Daejeon, 34036 Republic of Korea; 8 Department of Materials Science and Engineering, Yonsei University, Seoul, 03722 Republic of Korea; 9 Center for Human-Oriented Triboelectric Energy Harvesting, Yonsei University, Seoul, 03722 Republic of Korea; 10 Institute for Convergence Research and Education in Advanced Technology, Yonsei University, Seoul, 03772 Republic of Korea; 11 Department of Battery Engineering, Yonsei University, 50, Yonsei-Ro, Seodaemun-Gu, Seoul, 03722 Republic of Korea

**Keywords:** Energy harvesting storage hybrids, Triboelectric nanogenerators, Supercapacitors, Frequency response, MXene

## Abstract

Energy harvesting storage hybrid devices have garnered considerable attention as self-rechargeable power sources for wireless and ubiquitous electronics. Triboelectric nanogenerators (TENGs), a common type of energy harvester, generate alternating current-based, irregular short pulses, posing a challenge for storing the generated electrical energy in energy storage systems that typically operate with direct current (DC)-based low-frequency response. In this study, we propose a new strategy that leverages high-frequency response to develop efficient chargeable TENG–supercapacitor (SC) hybrid devices. A high-frequency SC was fabricated using hollow-structured MXene electrode materials, resulting in a twofold increase in the charging efficiency of the hybrid device compared to a control SC made with conventional carbon electrode materials. For a systematic understanding, the electrochemical interplay between the TENGs and SCs was investigated as a function of the frequency characteristics of SCs (*f*_SC_) and the output pulse duration of TENGs (Δt_TENG_). Increasing the *f*_SC_·Δt_TENG_ enhanced the charging efficiency of the TENG–SC hybrid devices. This study highlights the importance of frequency response design in developing efficient chargeable TENG–SC hybrid devices.

**Graphical Abstract:**

**Supplementary Information:**

The online version contains supplementary material available at 10.1007/s40820-025-01714-3.

## Highlights

- A system-level strategy is presented to achieve high charging efficiency in triboelectric nanogenerator (TENG)-supercapacitor (SC) hybrid devices, with a focus on frequency response design.
- This study reveals that the high-frequency characteristics of SCs and the prolonged output pulse duration of TENGs are critical for achieving high charging efficiency.
- A three-dimensional hollow-structured MXene is synthesized as a high-frequency SC electrode material, demonstrating a twofold increase in charging efficiency compared to conventional SCs.

**Supplementary Information:**

The online version contains supplementary material available at 10.1007/s40820-025-01714-3.

## Graphical abstract

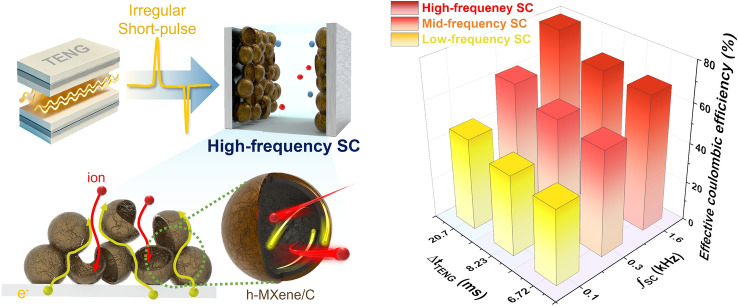

## Introduction

The escalating proliferation of wireless and ubiquitous electronic devices has spurred the development of advanced energy harvesters to provide autonomous mobile power sources, eliminating the need for external electrical charging. Among the various energy harvesting technologies, triboelectric nanogenerators (TENGs) have attracted significant attention due to their high-output power, ease of manufacturing, environmental friendliness, and high energy conversion efficiency [1–11]. However, TENGs typically produce intermittent and low-power outputs (nW to μW) due to uncontrolled operating environments. Consequently, integrating TENGs with energy storage systems is essential to ensure a stable and sustainable power supply [7, 12–14].

Supercapacitors (SCs) have emerged as a promising energy storage system for integration with TENGs, attracting considerable interest due to their rapid charge/discharge capability, long cycle life, and simplicity in cell fabrication [15, 16]. Despite this interest, most previous studies on energy harvester–SC hybrid devices have primarily focused on enhancing TENG performance [17, 18], with limited attention given to the electrochemical interplay between TENGs and SCs. Since conventional SCs are primarily designed for direct current (DC) operations exhibiting low-frequency responses (< 1 Hz), effectively storing the alternating current (AC)-based, irregular, and short-pulsed (i.e., high frequency of ~ kHz) energy from TENGs poses a significant challenge. In such short-pulsed current scenarios, most SCs may behave more like resistors than capacitors, leading to unwanted energy loss during the energy conversion/storage process [19, 20]. To address these limitations, previous research has introduced electrical engineering-based solutions, such as power management circuits [7, 21–23] and DC-driven TENGs [24–27]. However, these approaches have primarily focused on enhancing the apparent electrochemical performance of hybrid devices without a comprehensive understanding of energy storage efficiency and frequency characteristics.

Herein, we present a new system-level strategy focused on the frequency response design of TENG–SC hybrid devices for efficient storage of short-pulsed electric energy. Unlike previous studies that have primarily focused on optimizing TENG output or energy storage efficiency independently, our work, to the best of our knowledge, is the first to establish a direct correlation between the electrochemical characteristics of SCs and the frequency-dependent charging behavior of TENGs. While prior researches on TENG-SC systems have largely overlooked the role of SC frequency response, we demonstrate that the mismatch between the short-pulsed AC output of TENGs and the DC-based low-frequency operation of conventional SCs leads to inefficient energy storage.

**Fig. 1:**
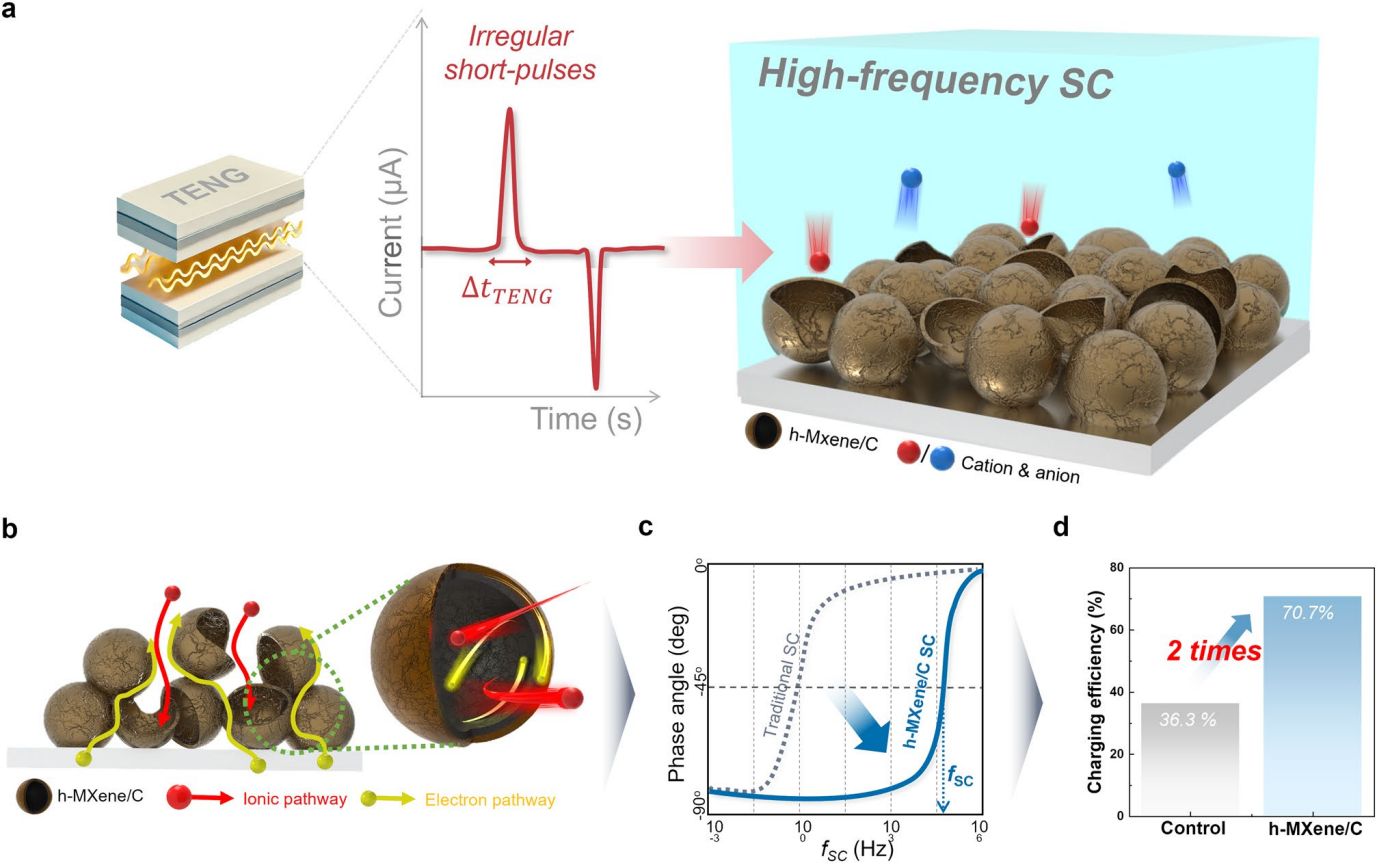
Schematic illustration showing high-frequency SC and TENG with exceptional charging efficiency. **a** Schematic illustration depicting the electrochemical interplay between TENG and high-frequency SCs. **b** Schematic representation of h-MXene/C and **c** its advantageous effect on frequency characteristics. **d** Comparison of charging efficiency between traditional SC and h-MXene/C SC

To address this issue, we explored the electrochemical interplay between TENGs and SCs, focusing particularly on the characteristic frequency of SCs (*f*_SC_) and the output pulse duration of TENGs (Δt_TENG_) (Fig. 1a). By introducing a high-frequency SC, we demonstrated that an enhanced frequency response enables more effective storage of short-pulse currents generated by TENGs, thereby improving the overall charging efficiency of the TENG–SC hybrid device. To elucidate the role of high-frequency SCs in enhancing charging efficiency, we developed three-dimensional (3D) hollow-structured MXene (h-MXene/C) as a high-frequency SC electrode material (Fig. 1b). The resultant high-frequency SC, characterized by its elevated frequency response (Fig. 1c), exhibited a twofold enhancement in charging efficiency compared to traditional SCs with conventional carbon electrodes (Fig. 1d). Notably, this enhancement in charging efficiency was achieved without any impedance matching between the SCs and TENGs, which is typically considered essential for efficient charging in harvesting﻿–storage hybrid configurations. Furthermore, we established a systematic correlation between *f*_SC_ and Δt_TENG_, which revealed that an increase in the product of *f*_SC_ and Δt_TENG_ facilitates the efficient storage of electricity generated by TENGs. This finding underscores the critical role of frequency response engineering in advancing next-generation self-powered energy storage technologies such as self-powered sensors [28–31] and wearable/biomedical device [32, 33], which face charging efficiency challenges.

## Experimental Section

### Materials Preparation

#### Synthesis of Polystyrene

Spherical polystyrene (PS) particles, with diameters of 2–3 μm, were synthesized using a previously described method [34]. Polyvinylpyrrolidone (2 g) was dissolved in a 250-mL two-neck flask, stirred, and heated to 70 °C in 160 mL of isopropanol with N_2_ purging. After 30 min, 20 g of styrene monomer and 0.2 g of 2,2-azobis(2-methylpropionitrile) were added, and the mixture was stirred for 20 h. The resulting white precipitate was collected via centrifugation and washed several times with distilled water (DIW) and ethanol. The final spherical PS particles were then dispersed in DIW.

#### Synthesis of MXene

The delaminated MXene, with the chemical formula Ti_3_C_2_T_x_, was synthesized by etching Al from Ti_3_AlC_2_. LiF (3 g) was dissolved in 9 M HCl solution (30 mL) and stirred for 30 min in a 100-mL polypropylene flask. Once the solution reached 50 °C, 3 g of Ti_3_AlC_2_ was gradually added and stirred for 24 h. The solution was then washed with DIW until the pH reached 6, using centrifugation. The MXene sediment was further centrifuged multiple times at 3500 rpm for 30 min to obtain the supernatant. This supernatant was repeatedly collected and centrifuged at 8500 rpm for 1 h to obtain the delaminated MXene. The delaminated MXene was then re-dispersed in DIW for electrode fabrication or freeze-dried for material characterization.

#### Preparation of h-MXene/C

The h-MXene/C was synthesized using spherical PS particles, with diameters of 2–3 μm, as templates. The 2D MXene nanosheets easily adhered to the PS spheres due to the electrostatic interactions between the positively charged PS and the negatively charged MXene, attributed to the surface functional groups. The MXene-coated PS spheres were then annealed at 500 °C for 1 h under an Ar atmosphere to remove the PS template, leaving a carbon layer on the inner surface of the h-MXene.

### Supercapacitor Fabrication

#### Fabrication of h-MXene/C SCs

The h-MXene/C SC electrodes were fabricated using a spray printing technique. The electrode inks were prepared by mixing a commercial PEDOT:PSS solution (PH1000, Clevios) with a solvent mixture of IPA (Duksan Co.), DIW, and dimethyl sulfoxide (DMSO, Samchun Co.). Next, h-MXene/C was added to the PEDOT: PSS solution, resulting in a composition ratio of h-MXene/C/PH1000/IPA/DIW/DMSO = 0.26/0.13/76.7/21/1.91 (w/w/w/w/w). After sonication for 30 min, the h-MXene/C SC inks were spray-printed onto preheated (75 °C) nickel current collectors using a commercial airbrush (LWA 266, Mr. Hobby). The spraying conditions were maintained at 15 psi of air pressure and a 10 cm distance between the nozzle and the substrate. The printed electrodes were then dried in a vacuum oven at 60 °C for 12 h. For the control SCs, electrode slurries were prepared with a composition ratio of carbon active material (Super P) to CMC binder of 7/3 (w/w). Control electrodes were fabricated by casting the electrode slurry onto a nickel current collector. Before cell assembly, the cast electrode slurry was dried at 60 °C for 2 h and vacuum-dried at 120 °C for 12 h. Both types of electrodes were assembled using a 2032-type coin cell (consisting of 180-μm glass fiber as a separator and [EMIM][TFSI] as the electrolyte). The overall capacitance of the h-MXene/C and control SCs was matched through a parallel connection.

#### Fabrication of Model SCs

The High-SC and Mid-SC electrodes were fabricated using a spray printing technique. To prepare the electrode inks for spray printing, a commercial PEDOT: PSS solution (PH1000, Clevios) was mixed with a solvent mixture composed of isopropyl alcohol (IPA, Duksan Co.), DIW, and DMSO. This mixture was stirred for 24 h. Subsequently, carbon black (Super P) was added to the solvent mixture and bath-sonicated for 30 min. The composition ratio of the electrode ink was Super P/PH1000/IPA/DIW/DMSO = 0.26/0.13/76.7/21/1.91 (w/w/w/w/w). The electrode inks were then sprayed onto a preheated (75 °C) nickel current collector using a commercial airbrush (LWA 266, Mr. Hobby). The spray conditions were maintained at an air pressure of 15 psi with a 10-cm gap between the nozzle and the substrate. The electrodes were subsequently dried in a vacuum oven at 60 °C for 12 h. For Low-SC electrodes, the electrode slurries were prepared with a composition ratio of Super P/carboxymethyl cellulose (CMC) = 7/3 (w/w) and were fabricated by casting the electrode slurry onto a nickel current collector. Before cell assembly, the cast electrode slurry was dried at 60 °C for 2 h and then vacuum-dried at 120 °C for 12 h. All model SC electrodes were assembled using 2032-type coin cells, which included a 180-μm glass fiber separator and [EMIM][TFSI] as the electrolyte.

### Characterization

#### Material and Electrochemical Characterization

The surface and cross-sectional morphologies of all components were investigated using field emission scanning electron microscopy (FE-SEM, Regulus 8100, Hitachi). The electronic conductivity of the electrodes was measured using a four-point probe measurement system (FPP-RS8, DASOLENG). The morphology of the h-MXene/C particles was observed using transmission electron microscopy (TEM, JEM-2100, JEOL). The electron energy loss spectroscopy (EELS) mapping was performed with a Cs-corrected high-resolution scanning transmission electron microscope (JEM-2200FS, JEOL). The h-MXene/C pore size was measured using a mercury porosimeter (PoreMaster 33, Anton Paar). Thermogravimetric data were obtained with a thermogravimetric analyzer (TGA 5500, TA Instruments). Surface analysis was conducted using X-ray photoelectron spectroscopy (XPS, ESCALAB 250, VG Scientific). The X-ray diffraction (XRD) patterns were obtained using an X-ray diffractometer (D/MAX-2500/PC, Rigaku). Raman spectra were measured using a Raman spectrometer (HEDA 250, WEVE). Visible and NIR spectra of all samples dispersed in DIW were measured using a UV/Vis spectrophotometer (Ubi-490, Microdigital) within the wavelength range of 400–1100 nm.

The electrochemical performance of the SCs was characterized using a potentiostat/galvanostat (VMP-300, Bio-Logic). The gravimetric capacitance was calculated from the CV profile using the following equation:

1 $${C}_{g} \left(F {g}^{-1}\right)=\frac{\int I dv (\text{mA }\times \text{V})}{2 \times v \left(\text{mV }{\text{s}}^{-1}\right)\times \Delta V \left(\text{V}\right) \times m (\text{g})}$$

where ${C}_{g}$, *I*, *ν*, Δ*V*, *m* are the gravimetric capacitance (F g^−1^), corresponding current (mA), scan rate (mV s^−1^), potential window (V), and mass of active material in the two electrodes (g^−1^). Electrochemical impedance spectroscopy (EIS) measurements were taken in the frequency range from 10^−2^ to 10^5^ Hz at an applied amplitude of 10 mV. In this context, the complex model of the frequency-dependent capacitance $C^{\prime } \left(\omega \right)$, ${C}^{\prime\prime}\left(\omega \right)$, and $C\left(\omega \right)$ is considered as follows:

2 $$C(\omega ) = C^{\prime } (\omega ) - jC^{{\prime \prime }} (\omega )$$

3 $${C}^{\prime}\left(\omega \right)=\frac{-{Z}^{\prime\prime} \left(\omega \right)}{{\omega \left|Z\left(\omega \right)\right|}^{2}}$$

4 $${C}^{\prime\prime}\left(\omega \right)=\frac{{Z}^{\prime}\left(\omega \right)}{{\omega \left|Z\left(\omega \right)\right|}^{2}}$$

5 $$\omega =2\pi f$$

6 $${C}_{A}\left(\omega \right)= \frac{1}{\omega {Z}^{\prime\prime}\left(\omega \right)A}$$

where $C\left(\omega \right)$ is complex impedance, ${C}^{\prime}\left(\omega \right)$ and ${C}^{\prime\prime}\left(\omega \right)$ are the real and imaginary parts of capacitance, ${Z}^{\prime}\left(\omega \right)$ and ${Z}^{\prime\prime}\left(\omega \right)$ are real and imaginary parts of impedance, and *f* is the frequency. ${C}_{A}\left(\omega \right)$ is the frequency-dependent areal capacitance calculated from the EIS results, and *A* is the surface area of the electrode.

#### Characterization of Electrochemical Performance of TENG–SC Hybrid Systems

The output performance of the TENGs was evaluated using an electrometer (Model 6514, Keithley) and an oscilloscope (TBS2072B, Tektronix). The electrochemical characteristics of the TENG–SC hybrid system were assessed with a potentiostat/galvanostat (VMP-300, Bio-Logic). A vibration system (LW126.151-9, LabWorks Inc.) and a custom-made rotator controlled the external pushing forces applied to the TENGs during energy harvesting.

#### Characterization of AC Line-Filtering Performance of the h-MXene/C SCs

For the AC line-filtering test, input signals were generated and amplified by an arbitrary function generator (AFG-2125, GW Instek) and an amplifier (PA-151, LabWorks Inc.). The resulting outputs were captured using an oscilloscope (TBS2072B, Tektronix).

## Results and Discussion

### Achieving Higher-Frequency Response of SC by Hollow MXene/C Electrode Materials

**Fig. 2:**
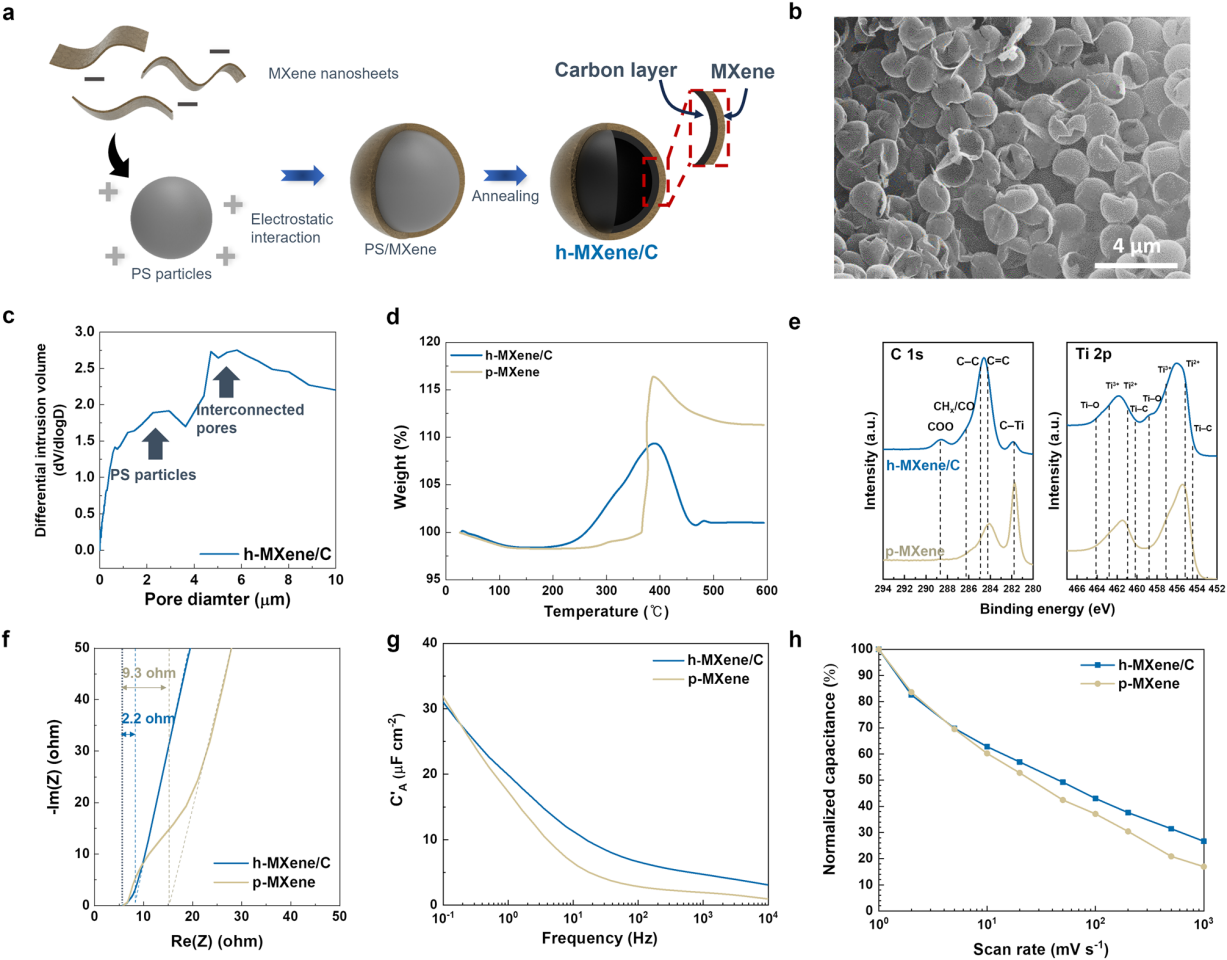
Achieving higher-frequency response of SC by hollow MXene/C electrode materials. **a** Schematic of the synthesis process for h-MXene/C particles. **b** SEM image of the h-MXene/C particles. **c** Pore size distribution of the h-MXene/C particles, determined by mercury intrusion porosimetry. **d** TGA profiles of the h-MXene/C and p-MXene in air. **e** XPS spectra (C 1*s* and Ti 2*p*) of the h-MXene/C and p-MXene. **f** Nyquist plots for the h-MXene/C and p-MXene SCs. **g** Frequency-dependent areal capacitance of the h-MXene/C and p-MXene SCs. **h** Rate capability of the h-MXene/C and p-MXene SCs at various scan rates

The frequency response of SCs is influenced by various factors, including the electrical conductivity of electrode materials and the interfacial resistance between the electrodes and current collectors [19]. Notably, the pore structure of the electrodes and the ion diffusion kinetics within the porous electrodes significantly impact the frequency characteristics. To enhance both the frequency response and high capacitance of SCs, a new electrode material based on carbon-supported 3D hollow-structured MXene (h-MXene/C) particles was synthesized using PS microspheres as a pore-forming carbon source (Fig. 2a). The synthesis details of h-MXene/C are provided in the experimental section. The percolated hollow structure of the h-MXene/C was confirmed through SEM and TEM imaging (Figs. 2b and S1). Mercury porosimetry analysis estimated the pore size of the h-MXene/C to be approximately 2 μm (Fig. 2c). An open porous structure was also achieved by the fusion of adjacent PS particles, followed by a thermal decomposition step. During this process, PS particles coated with nanoscale MXene sheets underwent thermal decomposition at 500 °C, resulting in a carbon-supported, interconnected 3D hollow structure [35].

The presence of carbon in the h-MXene/C was confirmed through thermogravimetric analysis (TGA) (Fig. 2d). In an air atmosphere, pristine MXene (p-MXene) showed a rapid weight increase at ~ 370 °C due to oxidation, forming TiO_2_ [36]. Conversely, h-MXene/C exhibited a drastic weight loss above 400 °C, indicating the thermal decomposition of the carbon layer in the h-MXene/C. The existence of the carbon layer was further confirmed by X-ray photoelectron spectroscopy (XPS) (Fig. 2e). In the C 1*s* spectra, p-MXene exhibited characteristic peaks corresponding to C–Ti and C=C bonds [37]. By comparison, the h-MXene/C showed a reduction in the C–Ti peak, an increase in the C–C peak, and the emergence of new peaks corresponding to COO and CH_x_/CO bonds.

In the Ti 2*p* spectra, h-MXene/C shows characteristic peaks similar to those of p-MXene, indicating that the fundamental chemical state of MXene is largely preserved. However, a peak corresponding to Ti–O bonding appears around 458.9 eV, suggesting the formation of TiO_2_ due to partial oxidation of MXene during the heat treatment (Fig. 2e). The partial formation of TiO_2_ during the heat treatment was further confirmed by a minor peak around 28° in the XRD profiles (Fig. S2). Despite the partial formation of TiO_2_, heat treatment at 500 °C was conducted to ensure the carbonization of PS, which enhances structural rigidity and helps maintain the 3D hollow architecture (Fig. S3a, b). In contrast, heat treatment at 400 °C better preserves the intrinsic properties of MXene (Fig. S3c); however, the lower degree of carbonization results in PS remaining in a polymer-like state, which can reduce electrical conductivity (Fig. S3d) [38].

In the XRD pattern of h-MXene/C, the 002 peak appeared broad, indicating that the MXene within the 3D structure consists of only a few layers (Fig. S2). Additionally, Raman spectroscopy showed that the characteristic A₁_g_ and E_9_ modes, corresponding to Ti–C bonds in MXene, are clearly observed in p-MXene. In contrast, h-MXene/C exhibited a slight decrease in the intensity of the A₁_g_ mode peak, indicating partial oxidation of MXene (Fig. S4a). Despite this oxidation, the presence of D and G bands in h-MXene/C, which are absent in p-MXene, confirms the formation of a carbon layer in h-MXene/C. Furthermore, the structural integrity of the h-MXene/C composite was confirmed by TEM and EELS (Fig. S4b). These results suggest that the carbonization process successfully introduced a carbonaceous component, which enhanced the structural stability and electrochemical properties of h-MXene/C. These results confirmed the formation of a carbon layer resulting in h-MXene/C with percolated pores. The unique structural characteristics of h-MXene/C are expected to facilitate liquid electrolyte infiltration and promote rapid electron transport, thus enabling efficient energy storage at high frequencies.

To fabricate SC electrodes, h-MXene/C particles were mixed with poly(3,4-ethylenedioxythiophene):poly(styrene sulfonate) (PEDOT:PSS), which served as a conductive binder. The electrostatic interactions between polar moieties on the Ti_3_C_2_ surface (such as −OH and −F) and PEDOT chains are known to enhance electrical conductivity by altering the resonance structure of PEDOT [39, 40]. The analysis of visible and near-infrared (NIR) spectra (Fig. S5) demonstrated a shift in the characteristic peak of PEDOT: PSS from 784 to 801 nm, implying the transformation of the PEDOT structure from benzoid to quinoid [41], facilitated by electrostatic interactions with the h-MXene/C.

The electrochemical kinetics of the h-MXene/C SCs were examined through EIS analysis (Fig. 2f). The equivalent distributed resistance (EDR) of the h-MXene/C was estimated to be 2.2 Ω, compared to the p-MXene, which exhibited a higher EDR of 9.3 Ω. These results highlight the significant role of the interconnected porous structure of the h-MXene/C in enhancing ion migration, as opposed to the densely stacked layered structure of the p-MXene (Fig. S6). Owing to this structural advantage, the h-MXene/C demonstrated higher areal capacitance than the p-MXene (Fig. 2g), which became more pronounced at higher frequencies. Furthermore, cyclic voltammetry (CV) analysis revealed the superior rate capability of the h-MXene/C compared to the p-MXene (Figs. 2h and S7), confirming the enhanced electrochemical kinetics afforded by its 3D hollow structure. Meanwhile, the galvanostatic charge–discharge (GCD) profiles of the h-MXene/C exhibited linear behavior over a wide range of current densities (1.0 to 10 μA cm^−2^), indicating negligible pseudo-capacitive reactions (Fig. S8a). The h-MXene/C also demonstrated stable capacitance retention (Fig. S8b), fulfilling a crucial requirement for TENG–SC hybrid devices operating under frequent and rapid charge–discharge cycles.

### Enabling Efficient Chargeable h-MXene/C SC-TENG Hybrid Devices

**Fig. 3:**
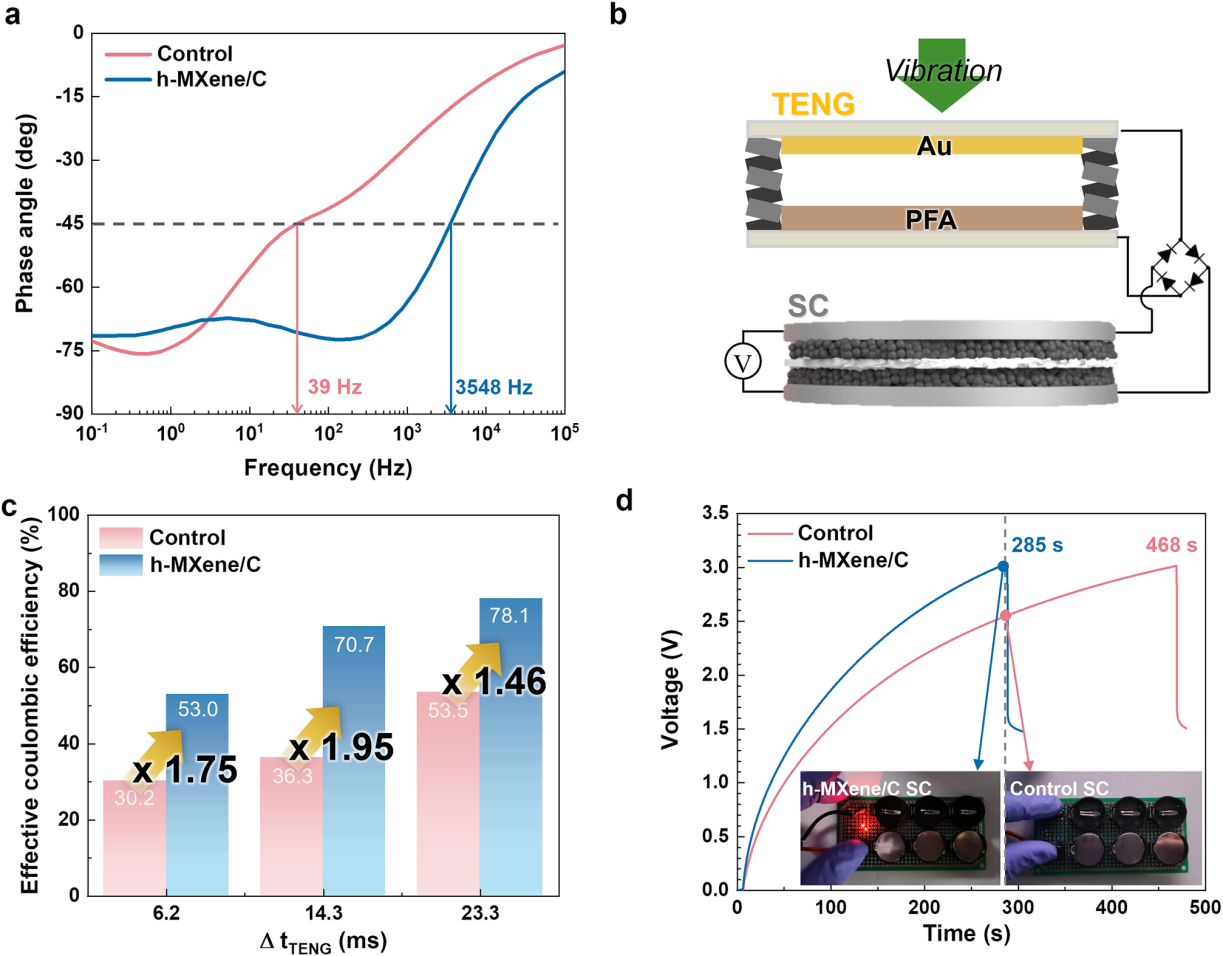
Enabling efficient chargeable h-MXene/C SC–TENG hybrid devices. **a** Bode plots comparing the h-MXene/C SC with a conventional carbon-based control SC. **b** Schematic of the experimental setup for the TENG–SC hybrid device. **c** Effective Coulombic efficiency of TENG–SC hybrid devices (h-MXene/C SC vs. control SC) as a function of Δt_TENG_. **d** Real-time voltage profiles for TENG–SC hybrid devices during the process of illuminating an LED. The inset shows the TENG–SC hybrid devices (h-MXene/C SC vs. control SC) after 285 s of charging time

The frequency characteristics of the h-MXene/C SC were investigated using EIS analysis (Fig. 3a). The *f*_SC_, defined as the frequency at a phase angle of −45°, is a critical metric for identifying the transition between capacitive and resistive behavior in SCs [19, 20]. The h-MXene/C SC exhibited a *f*_SC_ = 3548 Hz, significantly higher than that of the control SC (Fig. S9) with conventional carbon electrode materials (*f*_SC_ = 39 Hz). Additionally, the relaxation time for the h-MXene/C SC (0.38 ms) was considerably shorter than that of the control SC (128 ms) (Fig. S10), underscoring the faster electrochemical kinetics of the h-MXene/C SC.

To evaluate the improvement in charging performance, the h-MXene/C SCs were charged using a TENG comprising a gold positive electrode and a perfluoroalkoxy alkane (PFA) negative electrode (Fig. S11). Note that all the TENG–SC charging tests were conducted without any impedance matching and electrical circuit design, which are commonly employed to enhance charging efficiency [7, 22, 42–45]. An AC output generated by the TENG with varying vibration frequencies of 3, 5, and 7 Hz was rectified via a bridge diode (Fig. 3b).

Detailed electrochemical performance of the TENG is provided in Figs. S12 and S13. Due to the faster electrochemical kinetics, the h-MXene/C SC demonstrated a rapid increase in charge storage compared to the control sample (Fig. S14) and improved effective Coulombic efficiencies (*η*) (Fig. 3c). The details of *η* are discussed in the next section (see Eq. 7). The h-MXene/C SC exhibited enhancements in the effective Coulombic efficiencies of 53.0%, 70.7%, and 78.1% which are 1.75, 1.95, and 1.46 times higher than the control SC at vibration frequencies of 3, 5, and 7 Hz, respectively. The enhanced effective Coulombic efficiency of h-MXene/C was maintained over a wide temperature range (25 to 70 °C) (Fig. S15a, b), but was decreased in high humidity environments due to TENG degradation (Fig. S15c, d). The high charging efficiency of the h-MXene/C SC–TENG hybrid device was further demonstrated by powering a light-emitting diode (LED), where three h-MXene/C SC unit cells were connected in series (Fig. S16). As shown in Fig. 3d, the h-MXene/C halved the time required to power the LED compared to the control SC.

This beneficial effect of the h-MXene/C SC was further emphasized when paired with various types of TENGs. A high-output rotational TENG (rTENG), known for delivering higher output voltages and currents compared to the previously mentioned pushing-type TENGs [46], was fabricated. The h-MXene/C SC–rTENG hybrid device reduced the charging time by approximately 13.6% compared to the control SC–rTENG system (Fig. S17).

To demonstrate the material versatility of the frequency response design, we further investigated the effective Coulombic efficiency of the TENG–SC hybrid device using various high-frequency SC materials with an *f*_SC_ exceeding 1 kHz [47–49]. As shown in Fig. S18 and Table S1, a higher *f*_SC_ consistently led to an increase in effective Coulombic efficiency in the TENG–SC hybrid device, with all high-frequency SCs exhibiting improved efficiency compared to the control SC. This strong correlation confirms that the frequency response design is a fundamental and universal strategy for enhancing the charging efficiency of TENG–SC hybrid devices, regardless of the specific electrode material used.

Furthermore, we explored the potential application of the h-MXene/C SC in AC line-filters. The h-MXene/C SC effectively smoothed 60 Hz AC voltages, demonstrating its superior frequency characteristics (Fig. S19). Detailed experimental results for the AC line-filter are discussed in Note S1.

### Regulating the Frequency Response of SCs and Output Pulse Duration of TENGs for Efficient Storage of Short-Pulsed Energy Using Model SCs

**Fig. 4:**
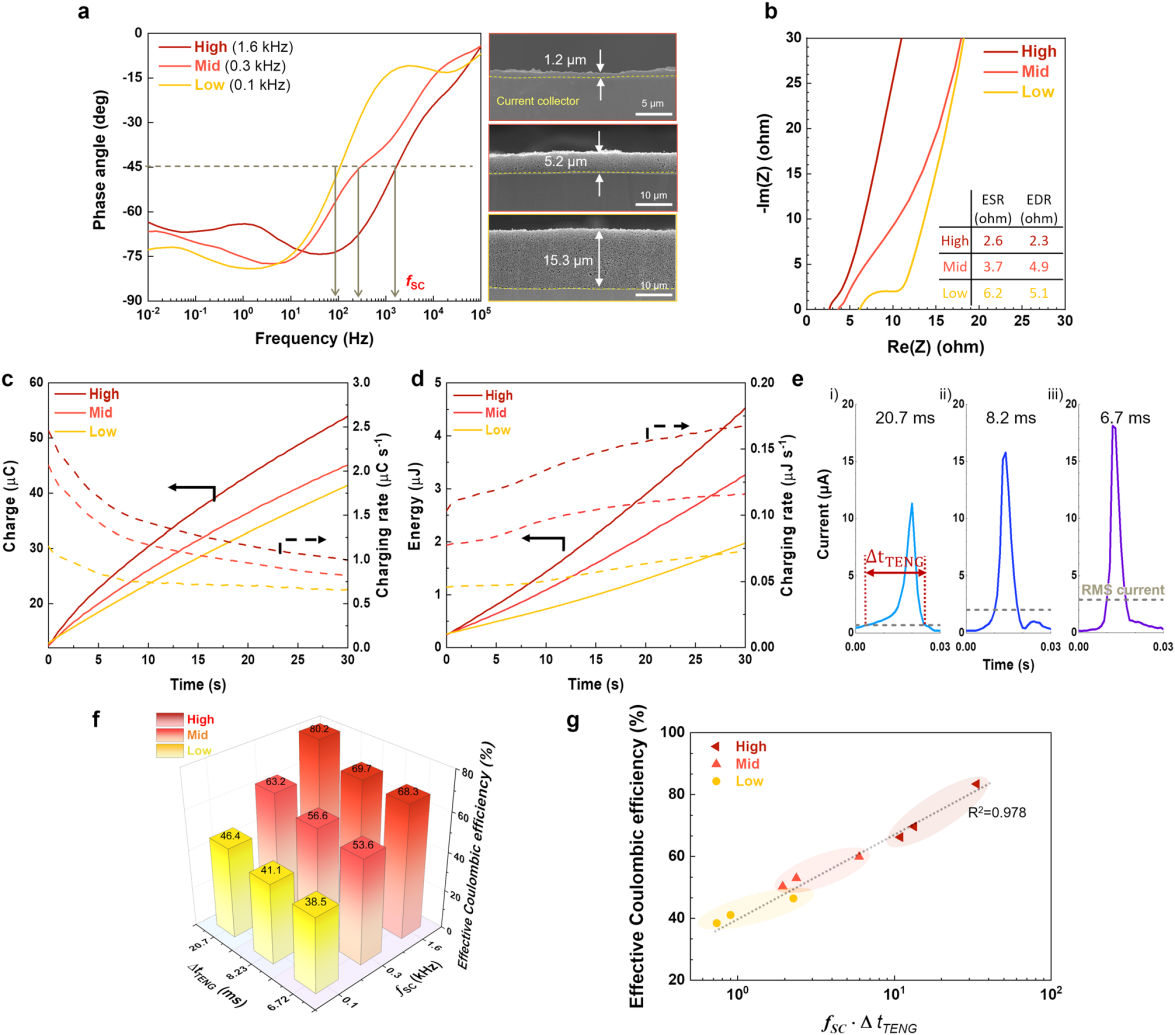
Regulating the frequency response of SCs and output pulse duration of TENGs for efficient storage of short-pulsed energy using model SCs. **a** Bode plots of the model SCs, with cross-sectional SEM images of their electrodes shown on the right. **b** Nyquist plots of the model SCs. **c** In situ monitoring of stored charge and charging rate in the model SCs as a function of charging time. **d** In situ monitoring of stored energy and charging rate in the model SCs as a function of charging time.** e** Output current waveforms of the TENG at various vibration frequencies and corresponding Δt_TENG_ values. **f** Effective Coulombic efficiency (*η*) of the model SCs as a function of Δt_TENG_ and *f*_SC_. **g** Comparison of effective Coulombic efficiency of TENG–SC hybrid devices as a function of *f*_SC_∙Δt_TENG_. All effective Coulombic efficiency values were measured after 10 s of TENG charging

To systematically understand the relationship between the frequency characteristics of SCs and charging efficiency, we examined the effect of *f*_SC_ and Δt_TENG_ on the charging efficiency of TENG–SC hybrid devices. Various model SCs with different frequency characteristics were fabricated by adjusting the electrical conductivity, thickness, and porosity of the electrodes (Fig. 4a and Table S2). Detailed fabrication processes for the model SCs are described in Note S2. These model SCs were classified according to their *f*_SC_ values (Fig. 4a): high-frequency SC (High-SC, *f*_SC_ = 1.6 kHz), medium-frequency SC (Mid-SC, 0.3 kHz), and low-frequency SC (Low-SC, 0.1 kHz). Analysis of Nyquist plots revealed that the High-SC had a low equivalent series resistance (ESR) of 2.6 Ω cm^−2^ and low EDR of 2.3 Ω cm^−2^, which were attributed to its highest electrical conductivity of 73.3 S cm^−1^ and thin electrode (thickness = 1.2 μm) (Fig. 4b and Table S2). This *f*_SC_ behavior was further confirmed through the analysis of complex frequency-dependent capacitances. The relaxation time constants (*τ*_0_), defined as the minimum time required to discharge all the energy from the SC with an efficiency greater than 50% of its maximum value [50, 51], were estimated to be 1.2 ms (High-SC), 12 ms (Mid-SC), and 18 ms (Low-SC), as shown in Fig. S20. To systematically compare the charging efficiencies between the different hybrid devices, the capacitances of the SCs were made identical by connecting the model SCs in parallel, which did not critically affect the *f*_SC_ values (Fig. S21 and Table S3) compared to the control SC.

Subsequently, these model SCs were charged using the same TENG device depicted in Fig. 3b at a vibration frequency of 3 Hz. The charging dynamics of the model SCs were investigated, focusing on in situ monitoring of voltage (V) and charge (Q) as a function of charging time (Fig. 4c). Notably, the High-SC, with a higher *f*_SC_, exhibited a faster charging rate compared to the SCs with lower *f*_SC_, leading to a rapid increase in stored energy (= V × Q) (Fig. 4d). These findings highlight the significant role of *f*_SC_ in the charging efficiency of TENG–SC hybrid devices.

In addition to the previously discussed *f*_SC_ results, the impact of Δt_TENG_ on the charging efficiency of TENG–SC hybrid devices was also examined. For reliable pulse duration measurements, Δt_TENG_ was defined as the time during which the output current exceeds the root-mean-square (RMS) of the output currents (Fig. 4e). It is important to note that changes in vibration frequency result in variations in Δt_TENG_ based on the Maxwell displacement current equation (Fig. S22 and Note S3). The Δt_TENG_ values were adjusted to 21, 8.2, and 6.7 ms by altering the TENG vibration frequency (Fig. 4e). Modulating Δt_TENG_ led to variations in output voltage and current (Fig. S11) due to variations in vibration frequency, resulting in different output powers. This variability made it challenging to directly compare charging efficiencies at different *f*_TENG_ values. To address this, we introduced *η*, defined by Eq. 7:

7 $${\text{Effective}}\;{\text{Coulombic}}\;{\text{efficiency}}\left( \eta \right) = \frac{{{\text{Charge}}\;{\text{emitted}}\;{\text{from}}\;{\text{SC}}}}{{{\text{Charge}}\;{\text{generated}}\;{\text{from}}\;{\text{TENG}}}} = \frac{{\mathop \smallint \nolimits_{0}^{{t_{disch.} }} I_{SC,disch.} dt}}{{\mathop \smallint \nolimits_{0}^{{t_{ch.} }} I_{TENG} dt}}\times100\left( \% \right)$$

where *I*_TENG_ represents the current generated from the TENG,* t*_ch_ denotes the charging time, *I*_SC,disch_ denotes the galvanostatic discharge current of the SC, and *t*_disch_ denotes the discharging time of the SC. Details of the experimental flowchart illustrating the steps for calculating the effective Coulombic efficiency are presented in Fig. S23. The model SCs (High-, Mid-, and Low-SCs) were charged by TENGs with varying Δt_TENG_ (Fig. S24) and then galvanostatically discharged at a constant current. Figure 4f demonstrates that increasing Δt_TENG_ (6.7 → 20.7 ms) enhances *η*, indicating that longer pulse durations of TENGs are favorable for energy storage in SC. For example, *η* enhanced from 38.5% to 46.4% as Δt_TENG_ increased from 6.72 to 20.7 ms at an *f*_SC_ of 0.1 kHz. A similar trend was observed at different *f*_SC_ values, where *η* enhanced from 53.6% to 63.2% at 0.3 kHz and from 68.3% to 80.2% at 1.6 kHz. This mechanistic understanding of *f*_SC_ and Δt_TENG_ revealed a linear relationship between *η* and the product of *f*_SC_ and Δt_TENG_ on a logarithmic scale (Fig. 4g). Notably, higher *f*_SC_ and Δt_TENG_ values are preferred for efficiently storing short-pulsed electric energy in TENG–SC hybrid devices.

## Conclusions

We presented a high-frequency response design SC to enable efficient charging with TENG, grounded in a comprehensive analysis of the electrochemical interplay between TENGs and SCs. To achieve high-frequency SCs, we synthesized h-MXene/C as an electrode material. The h-MXene/C SC exhibited rapid frequency characteristics (*f*_SC_ = 3548 Hz vs. 39 Hz for the control SC), attributed to its distinctive structural feature, specifically a carbon-supported, percolated hollow structure. Consequently, the h-MXene/C SC demonstrated a twofold increase in charging efficiency compared to conventional SCs. We further investigated the charging efficiency of TENG–SC hybrid devices as a function of the SC characteristic frequencies and TENG output pulse duration. The parameter *f*_SC_⋅Δt_TENG_ emerged as a critical factor influencing charging efficiency. Higher *f*_SC_⋅Δt_TENG_ values were achieved by pairing high-frequency SCs with long-pulse-duration TENGs, thereby enhancing the charging efficiency of TENG–SC hybrid devices. Given the parametric relationship between *f*_SC_⋅Δt_TENG_ and charging efficiency, we suggest that this frequency–response design can be achieved not only with the h-MXene/C presented herein but also with other high-frequency supercapacitors such as those used in AC line-filtering applications. This frequency–response design paves the way for developing advanced TENG–SC hybrid devices and offers promising potential as a platform technology for self-powered electrochemical systems facing charging efficiency challenges.

## Supplementary Information

Below is the link to the electronic supplementary material.

Supplementary file1 (DOCX 5197 KB)
