# Supplementary material for: Pulse-Charging Energy Storage for Triboelectric Nanogenerator Based on Frequency Modulation
Source: Nanomicro Lett. 2025 Apr 10;17:210. doi: 10.1007/s40820-025-01714-3 (PMC11985731; doi:10.1007/s40820-025-01714-3)
Supplement: Supplementary file 1 — Supplementary file1 (DOCX 5197 KB) [file 40820_2025_1714_MOESM1_ESM.docx]

Supporting Information for

**Pulse-Charging Energy Storage for Triboelectric Nanogenerator Based on Frequency Modulation**

Kwon-Hyung Lee^1,2†^, Min-Gyun Kim^1†^, Woosuk Kang^3†^, Hyun-moon Park^4^, Youngmin Cho^4^, Jeongsoo Hong^5^, Tae-Hee Kim^2^, Seung-Hyeok Kim^1,6^, Seok-Kyu Cho^7^, Donghyeon Kang^8,9^, Sang-Woo Kim^8,9^*, Changshin Jo^3,5,10^*, and Sang-Young Lee^1,11^*

^1^ Department of Chemical and Biomolecular Engineering, Yonsei University, Seoul, 03722, Republic of Korea

^2^ Ulsan Advanced Energy Technology R&D Center, Korea Institute of Energy Research (KIER), Ulsan, 44776, Republic of Korea

^3^ Department of Battery Engineering, Pohang University of Science and Technology (POSTECH), Pohang, 37673, Republic of Korea

^4^ Research and Development Center, Energy-Mining LTD, Suwon, 16226, Republic of Korea

^5^ Department of Chemical Engineering, POSTECH, Pohang, 37673, Republic of Korea

^6^ Department of Battery and Chemical Engineering, Hanyang University ERICA, Gyeonggi, 15588, Republic of Korea

^7^ UBATT Inc., Daejeon, 34036, Republic of Korea.

^8^ Department of Materials Science & Engineering, Yonsei University, Seoul, 03722, Republic of Korea

^9^ Center for Human-Oriented Triboelectric Energy Harvesting, Yonsei University, Seoul, 03722, Republic of Korea

^10^ Institute for Convergence Research and Education in Advanced Technology, Yonsei University, Seoul, 03772, Republic of Korea

^11^ Department of Battery Engineering, Yonsei University, Seoul, 03722, Republic of Korea

^†^Kwon-Hyung Lee, Min-Gyun Kim, and Woosuk Kang contributed equally to this work.

*Corresponding author. E-mail: [syleek@yonsei.ac.kr](mailto:syleek@yonsei.ac.kr) (Sang-Young Lee); [kimsw1@yonsei.ac.kr](mailto:kimsw1@yonsei.ac.kr) (Sang-Woo Kim); [jochangshin@postech.ac.kr](mailto:jochangshin@postech.ac.kr) (Changshin Jo)

**Note S1**

Aluminum electrolytic capacitors (AECs) are currently employed in AC line-filters due to their high-frequency characteristics [S1]. However, AECs require large volumes because of their low specific capacitance, which poses limitations for practical applications. To overcome this challenge, various studies have focused on developing alternative high-frequency SCs [S2-S5]. In this study, the application of the h-MXene/C SC for AC line-filters was explored using the AC/DC converter circuit depicted in Figure S19a. The AC voltage, with a frequency of 60 Hz, was converted into a smooth waveform by the h-MXene/C SC, demonstrating line filtering performance akin to that of the AEC (Fig. S19b, c). Furthermore, the inset of Figure 19c demonstrates the superiority of h-MXene/C SC over the AEC in size reduction.

**Note S2**

The frequency characteristics of SCs are influenced by various factors, including the electrical conductivity of electrode materials and the interfacial resistance between the electrodes and current collectors [S1]. Notably, the pore structure of electrodes and the ion diffusion kinetics within the porous electrodes play a significant role in determining the frequency characteristics [S1, S6]. Consequently, many previous studies on high-frequency SCs have focused on employing 1D or 2D materials as electrode components, such as vertically oriented graphene, PEDOT, and MXene, to ensure large electrode pores for achieving low EDR [S2, S5, S7, S8]. Conversely, activated carbon, commonly used in conventional SCs, exhibits a low *f*_SC_ of less than 1 Hz due to its numerous micropores and high tortuosity [S1, S9].

In this study, we fabricated three model SCs with distinct *f*_SC_ using different electrodes. Conductive carbon (Super P) was chosen as the conductive electrode material, combined with two different binders: PEDOT:PSS as a conductive binder and CMC as a non-conductive binder, to modulate electronic conductivity. Two fabrication techniques, spray coating and slurry casting, were employed to adjust the electrode thickness and porous structure. Detailed specifications are provided in Table S2. For the High-SC, a mixture of conductive carbon particles and PEDOT:PSS binders was spray-coated onto a Ni current collector at a composition ratio of 2/1 (w/w), forming a thin electrode (1.2 μm) with high electron conductivity (73.3 S cm^−1^). This configuration resulted in the High-SC possessing high-frequency characteristics (*f*_SC_ = 1.6 kHz), attributable to the minimization of porous effects. Meanwhile, for the Mid-SC (*f*_SC_ = 0.3 kHz), the electrode thickness was increased to 5.2 μm to extend the ion path within the porous electrodes. For the Low-SC (*f*_SC_ = 0.1 kHz), a Super P/CMC slurry at a composition ratio of 7/3 (w/w) was slurry-cast onto a Ni current collector, resulting in an electrode with a thickness of 15.3 μm and an electronic conductivity of 4.7 S cm^−1^.

**Note S3**

TENGs generate AC output through the mechanisms of contact electrification and electrostatic induction when distinct materials interact and subsequently separate. The output of the triboelectric generator is determined using the Maxwell displacement current equation, which is given by:

$$J_{D}= \sigma_{t}\frac{\partial z}{\partial t} \frac{d_{1}\varepsilon_{0}/\varepsilon_{1}+d_{2}\varepsilon_{0}/\varepsilon_{2}}{\left[ d_{1}\varepsilon_{0}/\varepsilon_{1}+d_{2}\varepsilon_{0}/\varepsilon_{2}+z\left( t \right) \right]^{2}}$$

where $J_{D}$ represents the displacement current density, $\sigma_{t}$ is the surface charge denisty, $\frac{\partial z}{\partial t}$ denotes the contact-separation frequency, $d_{1}$ and $d_{2}$ are the thicknesses of the materials, $\varepsilon_{0}$ is the vacuum permittivity, $\varepsilon_{1}$ and $\varepsilon_{2}$ are the permittivities of the materials, and $z\left( t \right)$ is the separation distance between the materials. According to this formula, an increase in the vibration frequency leads to a higher TENG output current density as the charge transfer rate during electrostatic induction rises with increased contact and separation frequency. However, this also results in a shorter Δt_TENG_ because the time required for surface charge compensation due to electrostatic induction decreases. Therefore, although the RMS current at low vibration frequencies is less than that at high frequencies, the Δt_TENG_ at low frequencies is greater. To clarify the relationship between vibration frequency and Δt_TENG_, we monitored the output current at extremely low vibration frequencies (Fig. S25). At vibration frequencies of 0.1, 0.2, and 0.5 Hz, the Δt_TENG_ of the output currents were measured as 624, 331, and 118 ms, respectively, which are significantly larger than the experimental results shown in Figure 4e (vibration frequencies of 3, 5, and 7 Hz resulting in 20.7, 8.2, and 6.7 ms of Δt_TENG_, respectively).

**Supplementary Figures**

**
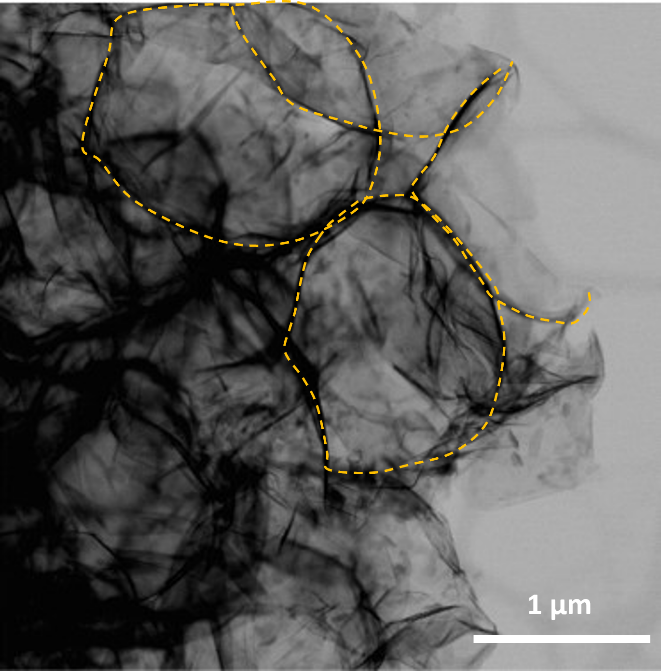
**

**Fig. S1** TEM images of the h-MXene/C particles


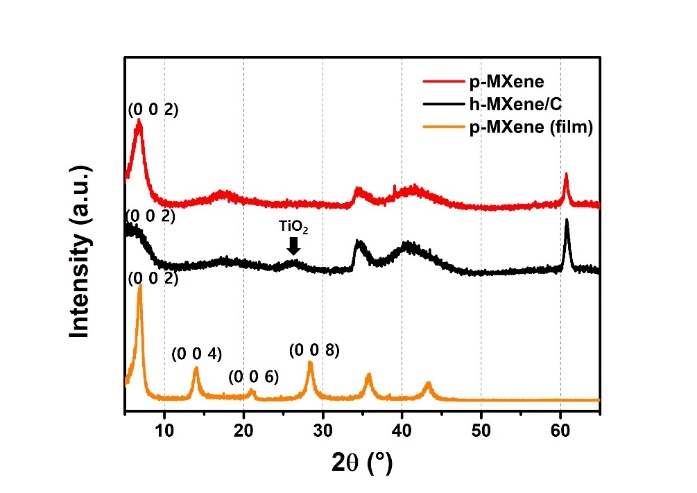


**Fig. S2** XRD pattern of p-MXene, h-MXene/C, p-MXene film. The broadening of 002 peak of h-MXene/C suggests a reduced degree of stacking, confirming that the MXene sheets were loosely packed and maintained a few-layered configuration within the 3D hollow architecture. In contrast, p-MXene, which was freeze-dried, shows a sharp 002 peak owing to the re-stacking during the drying process. The stronger intensity of the 002 peak is associated with the more pronounced stacking of MXene sheets, which is clearly observed in the p-MXene film.


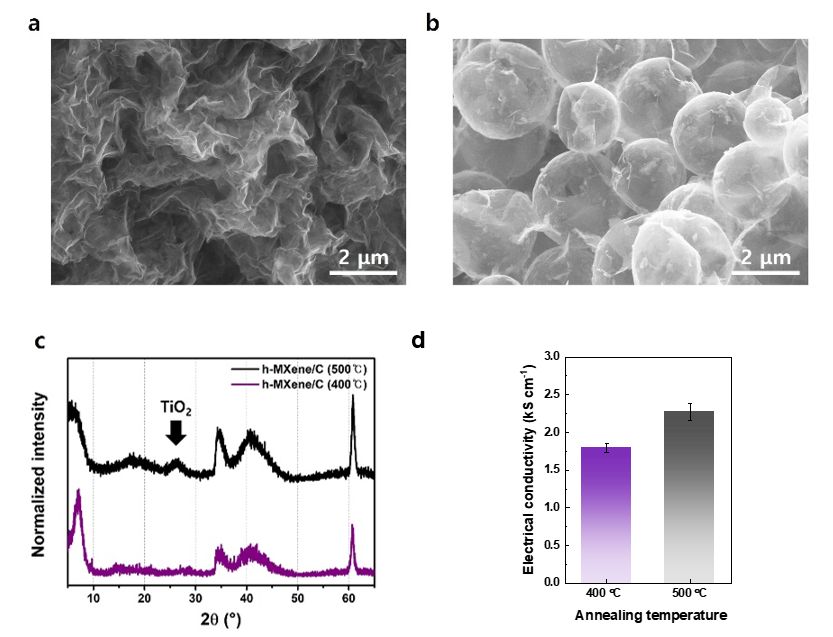


**Fig. S3** SEM images of h-MXene/C annealed at (**a**) 400℃ and (**b**) 500℃. Comparison of (**c**) XRD pattern and (**d**) electrical conductivity between the h-MXene/C annealed at 400℃ and 500℃


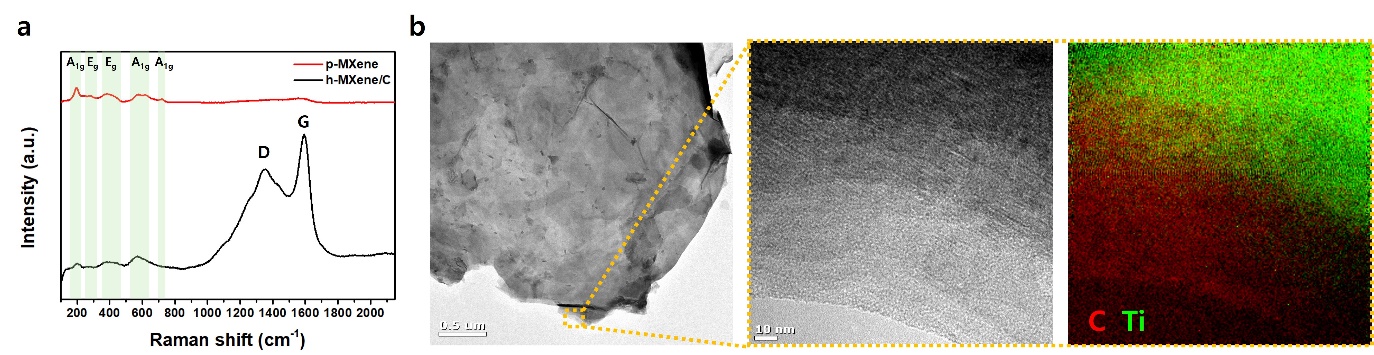


Fig. S4 (a) Raman spectra of p-MXene and h-MXene/C. (b) TEM image of h-MXene/C particle with different magnification and corresponding EELS mapping image


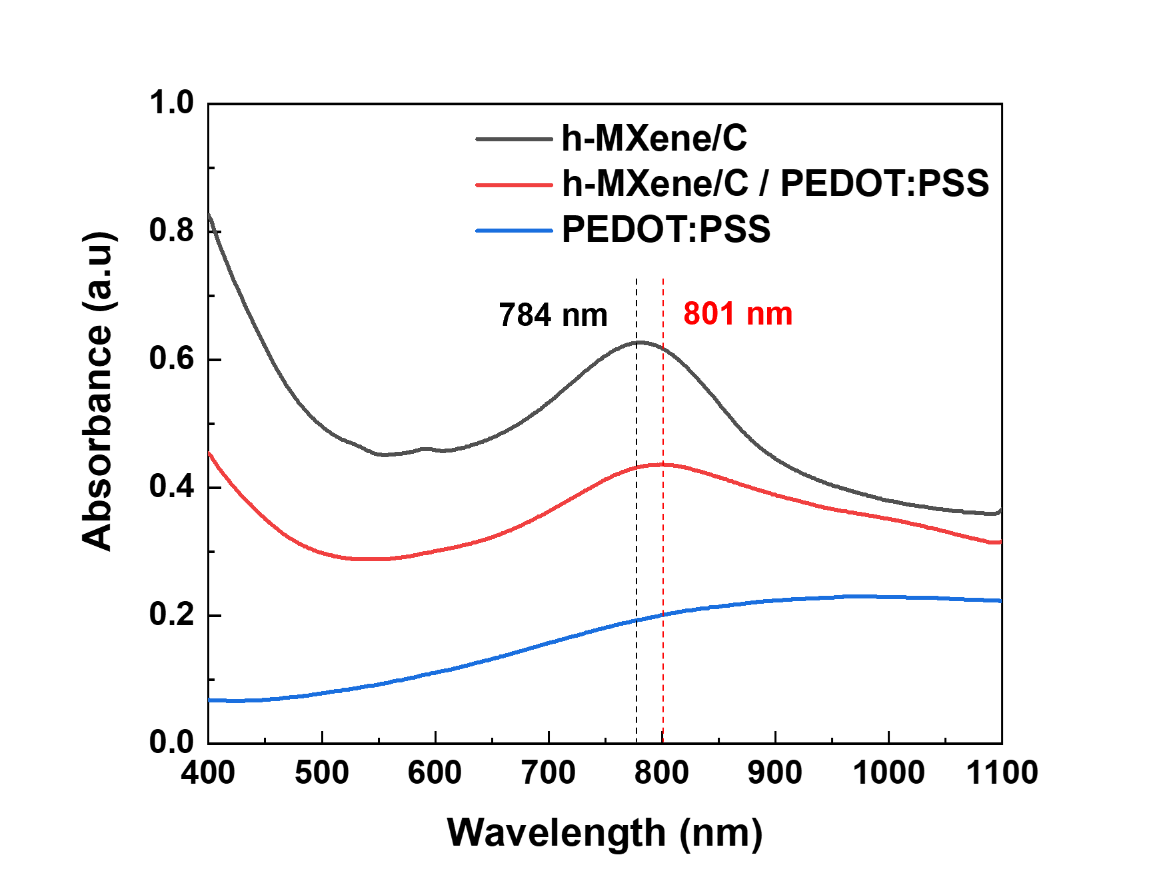


**Fig. S5** Visible and NIR spectra of aqueous solutions of h-MXene/C, PEDOT:PSS, and h-MXene-C/PEDOT:PSS


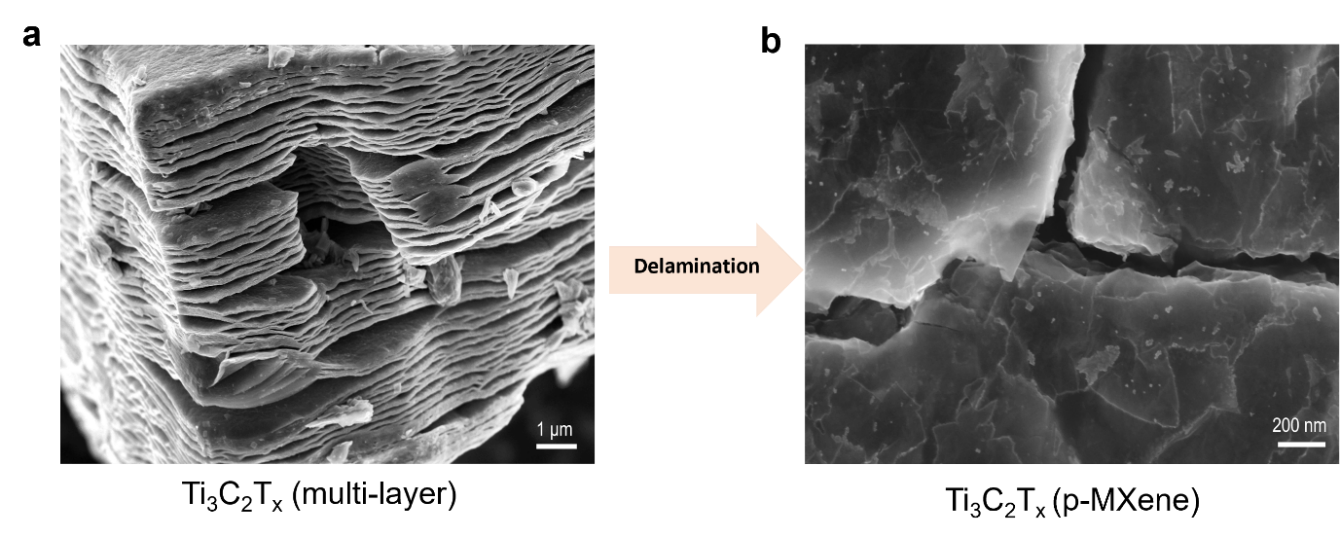


**Fig. S6** SEM images of (**a**) multi-layered MXene particles and (**b**) delaminated MXene particles after delamination (p-MXene)

**
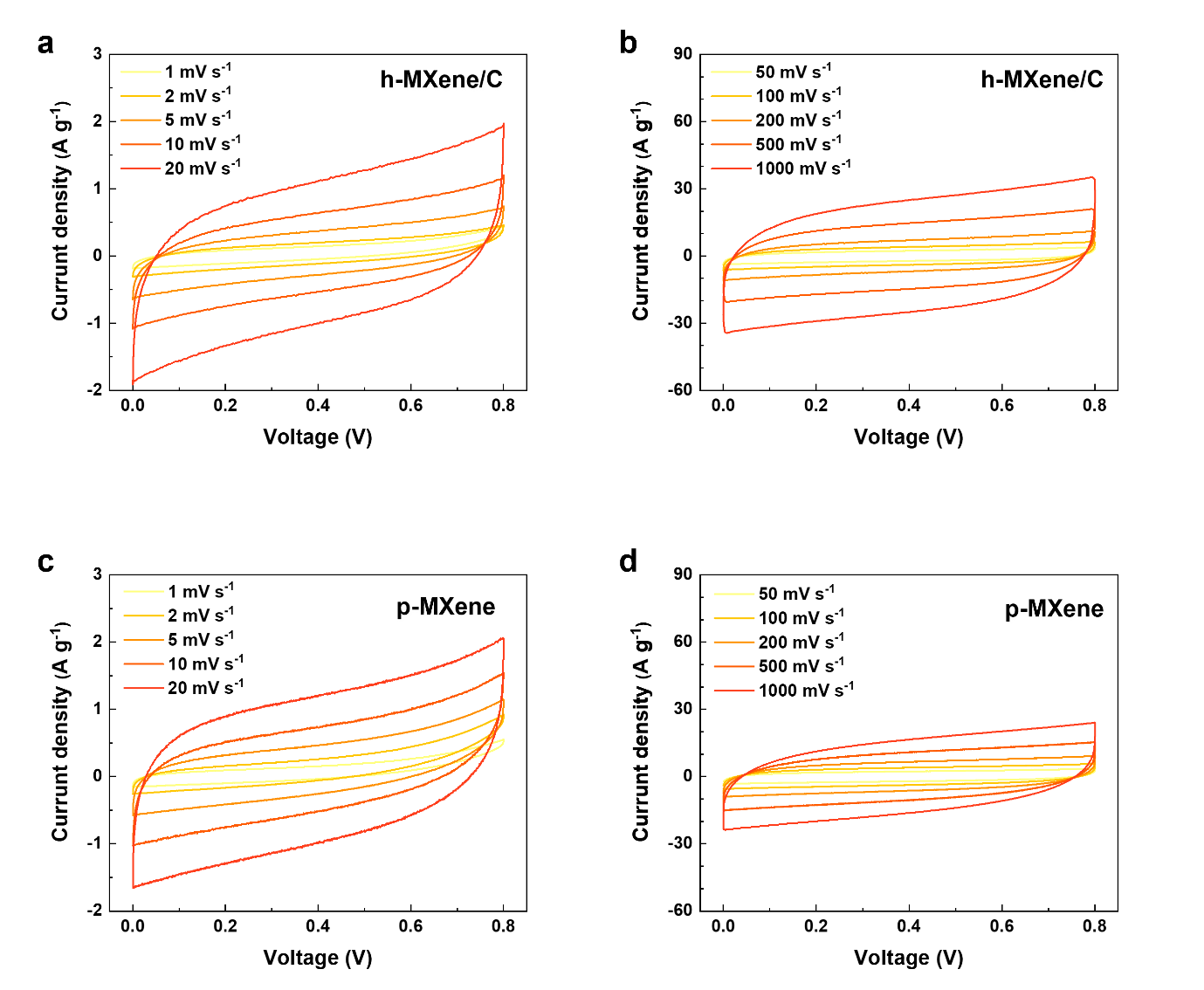
**

**Fig. S7** CV profiles (scan rate, 1 to 1000 mV s^−1^) of the (**a**, **b**) h-MXene/C SC and (**c**, **d**) p-MXene SC

**
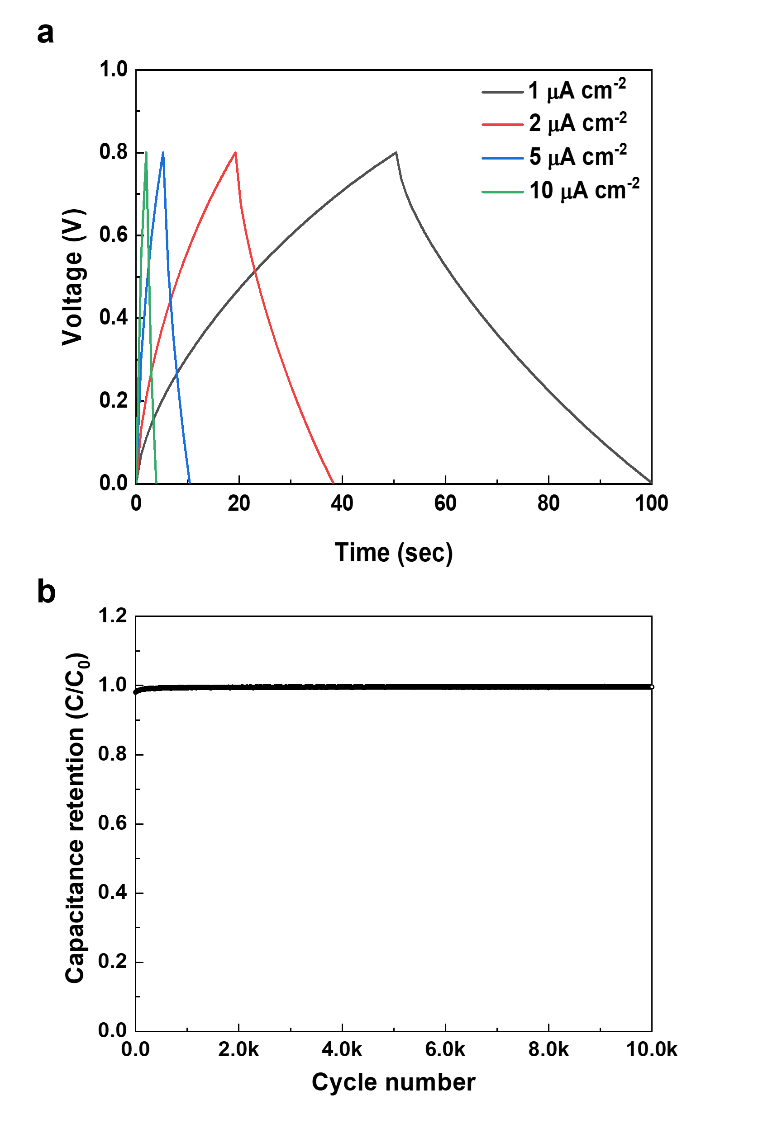
**

**Fig. S8** (**a)** GCD profiles (current density, 1 to 10 μA cm^−2^). (**b)** Capacitance retention of the h-MXene/C SCs


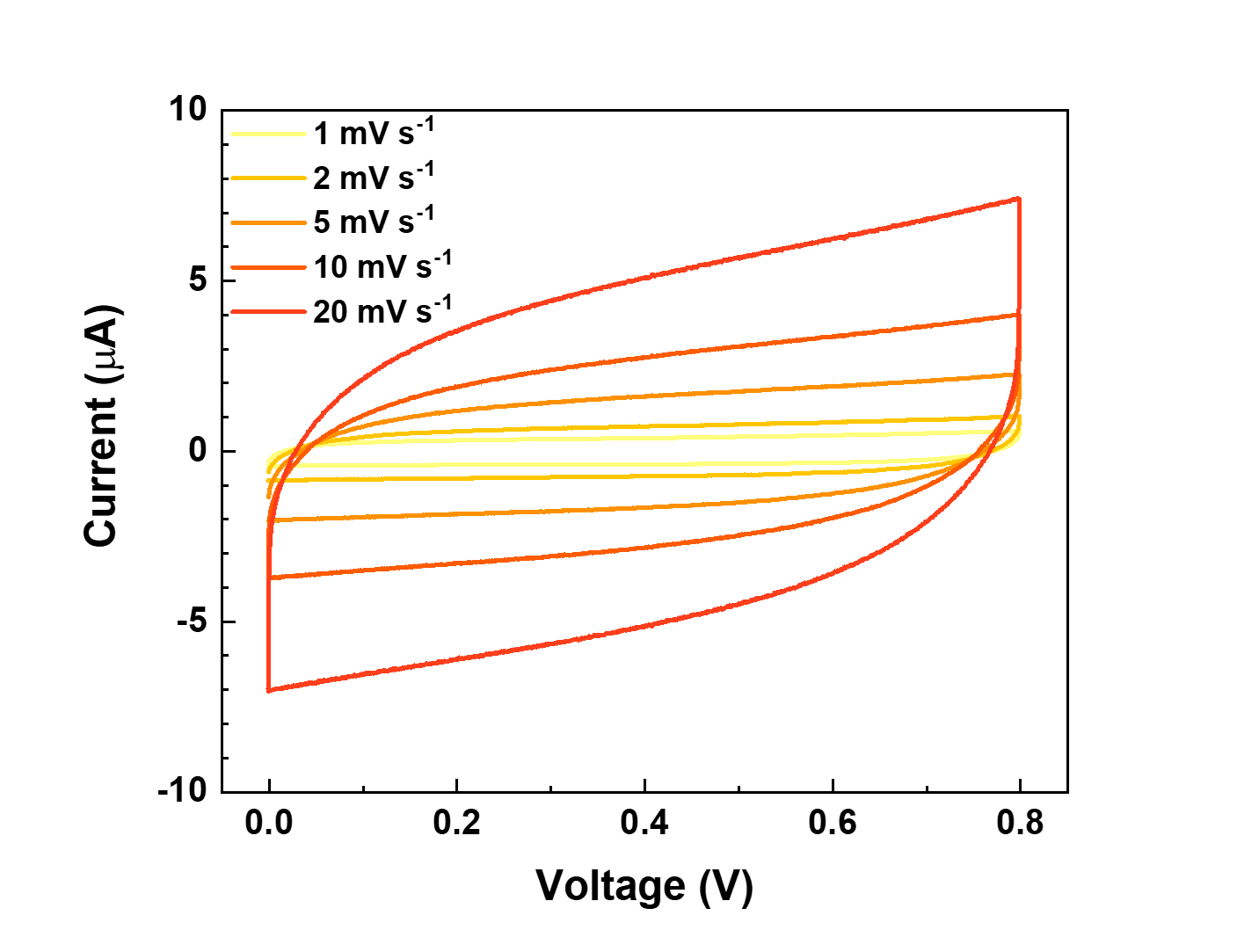


**Fig. S9** CV profiles of the control SC


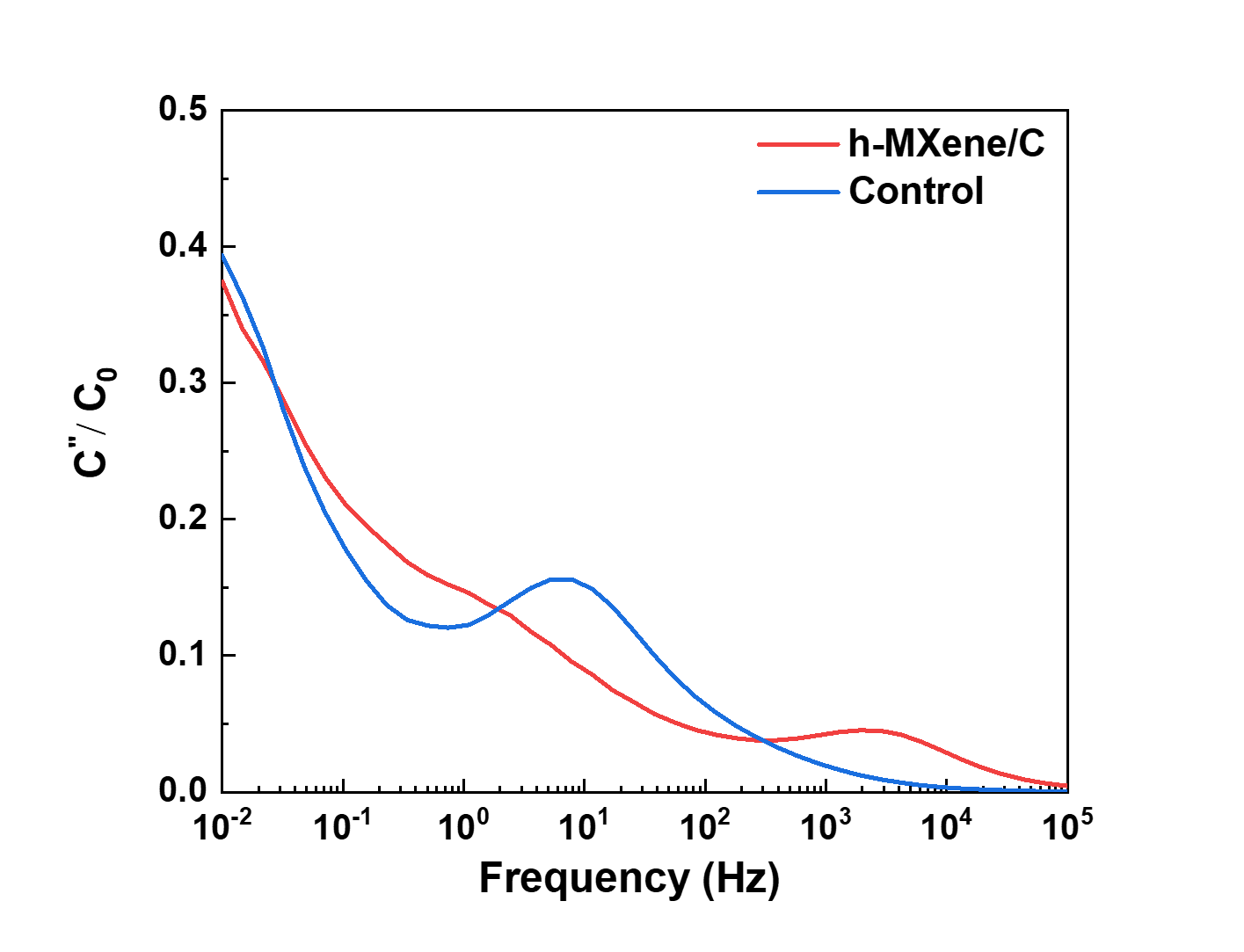


**Fig. S10** Imaginary part of the capacitance of the h-MXene/C SC and control SC as a function of frequency


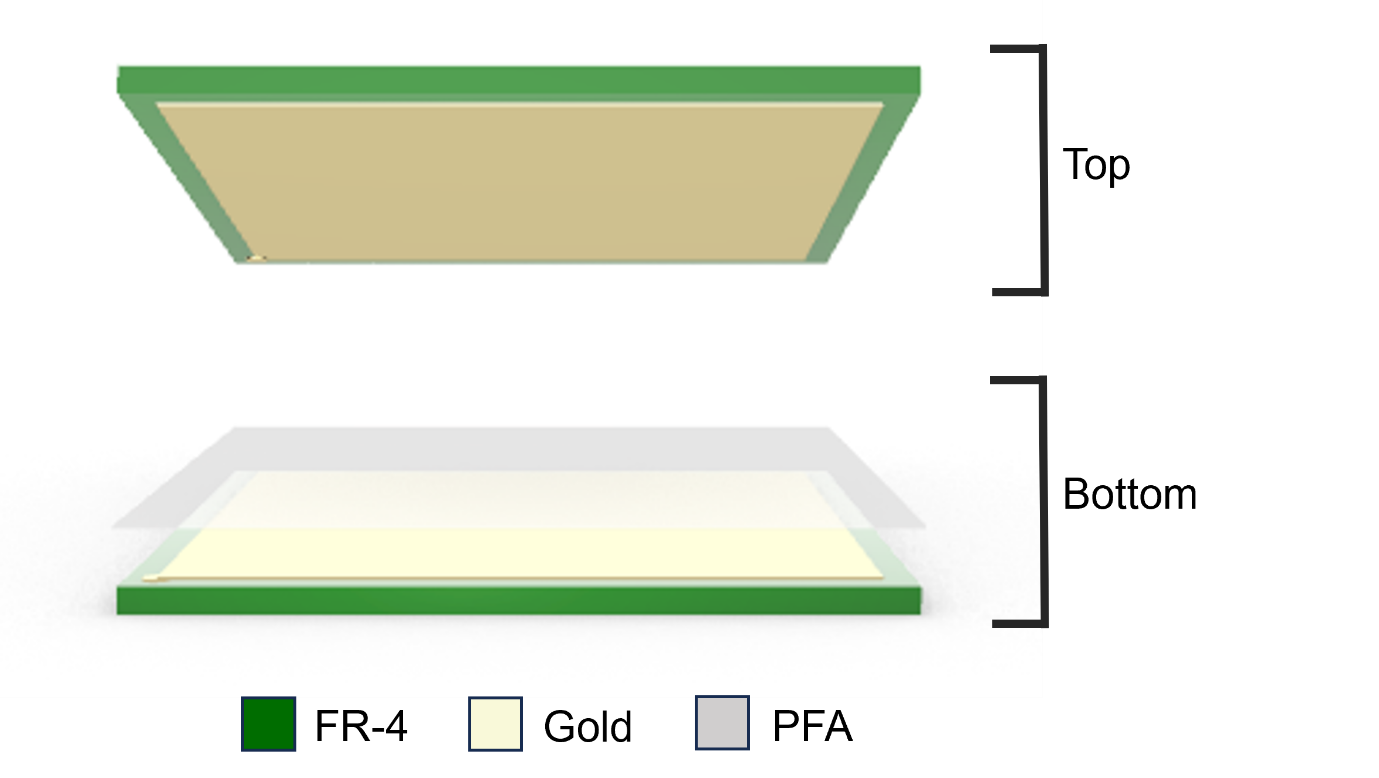


**Fig. S11** Schematic illustration showing the components of TENG

**
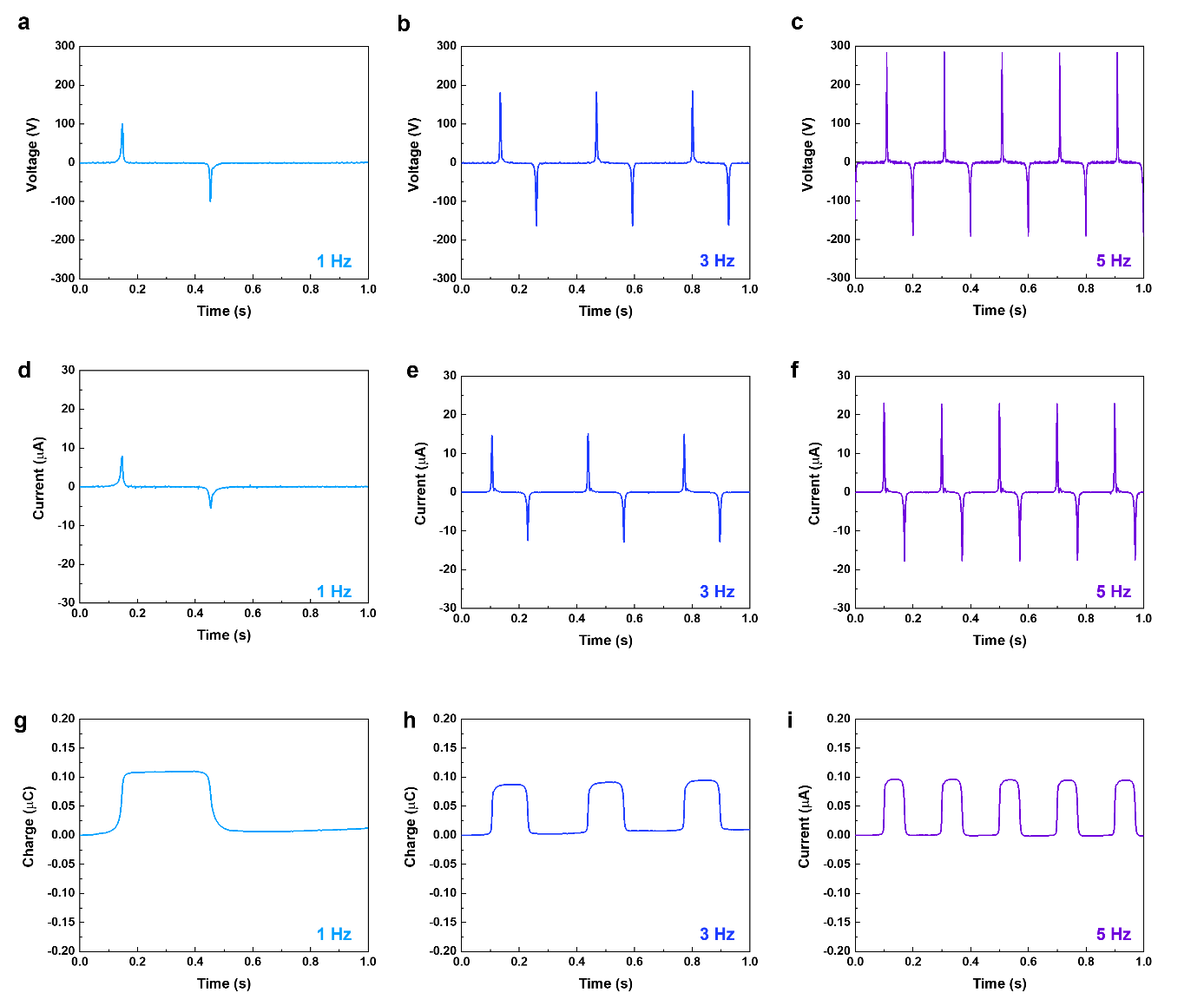
**

**Fig. S12** (**a**–**c**) Output voltage, (**d**–**f**) current waveforms (**g–i**) charge generation of the TENG at various vibration frequencies (1, 3, and 5 Hz, respectively)


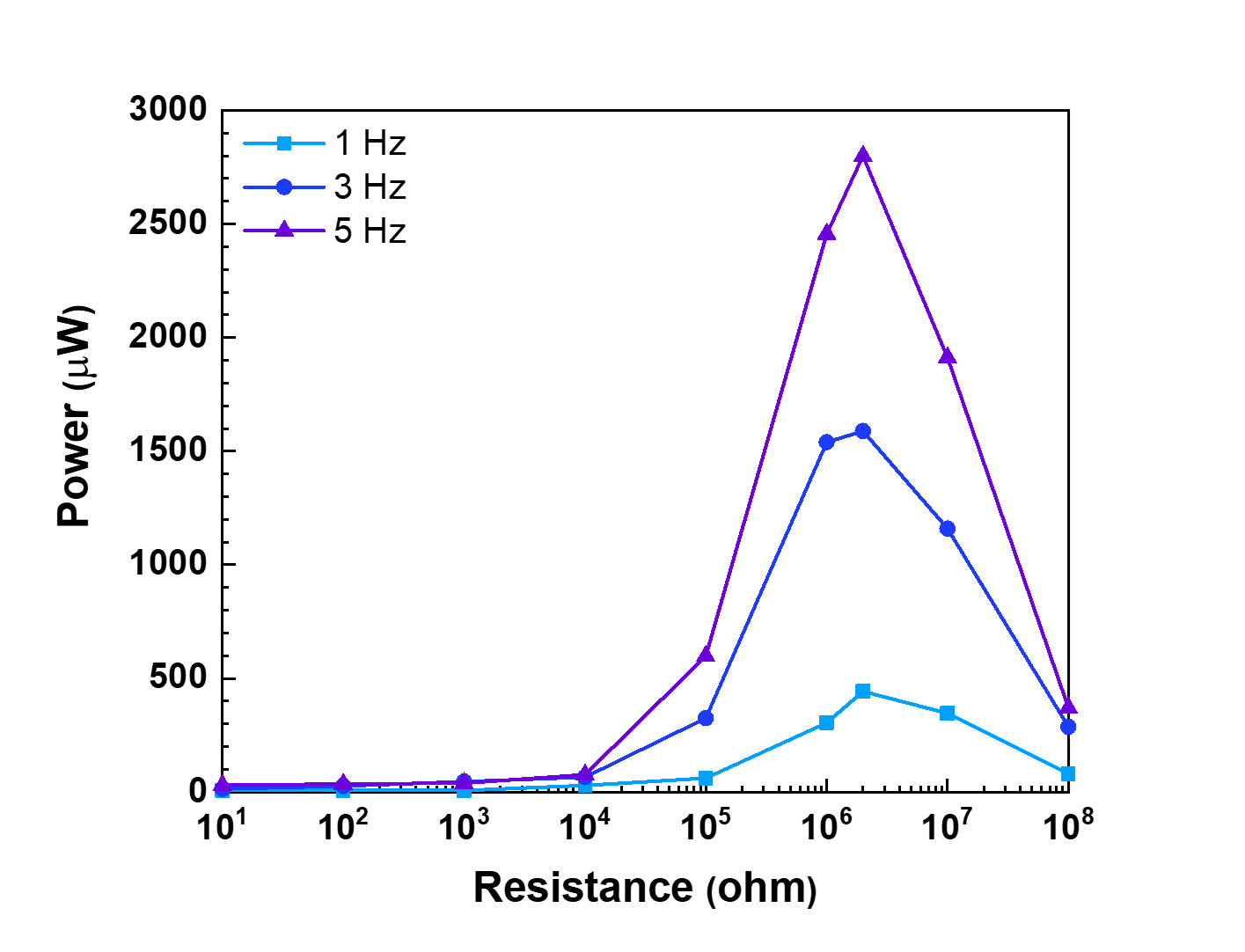


**Fig. S13** Output power performance of the TENG as a function of external load resistance at various vibration frequencies.


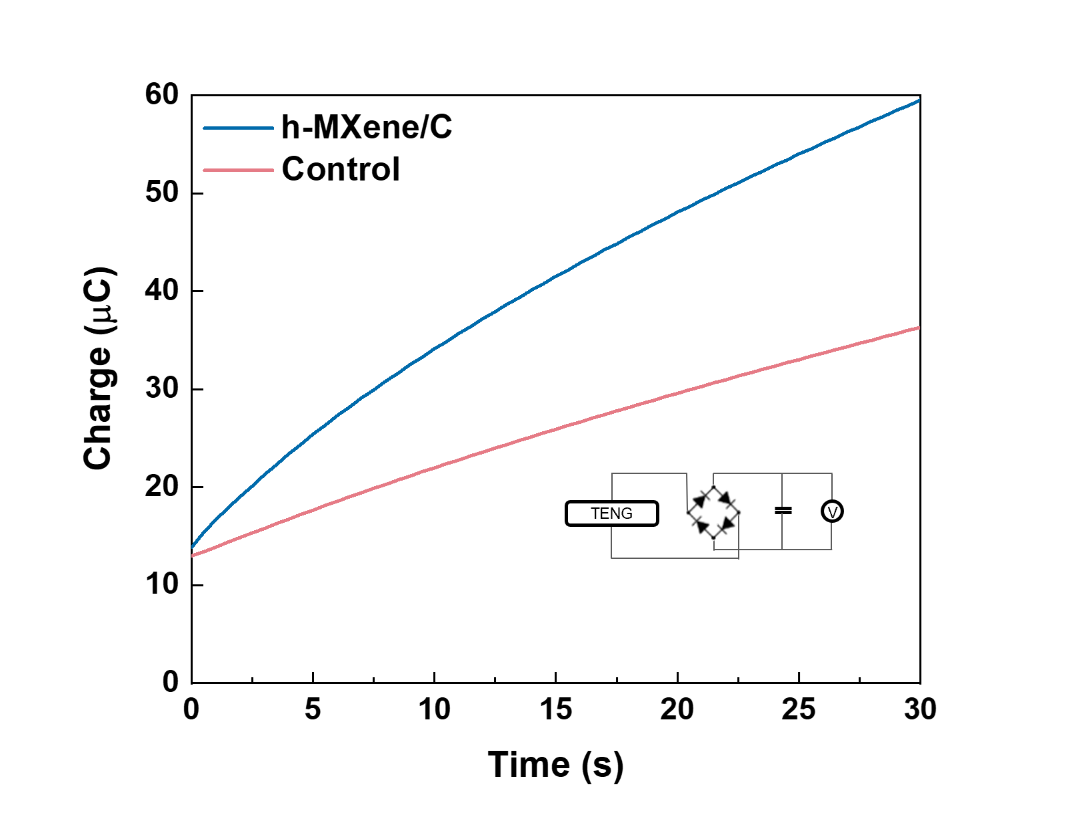


**Fig. S14** Real-time charge profiles stored in the h-MXene/C SC and the control SC during TENG charging

**
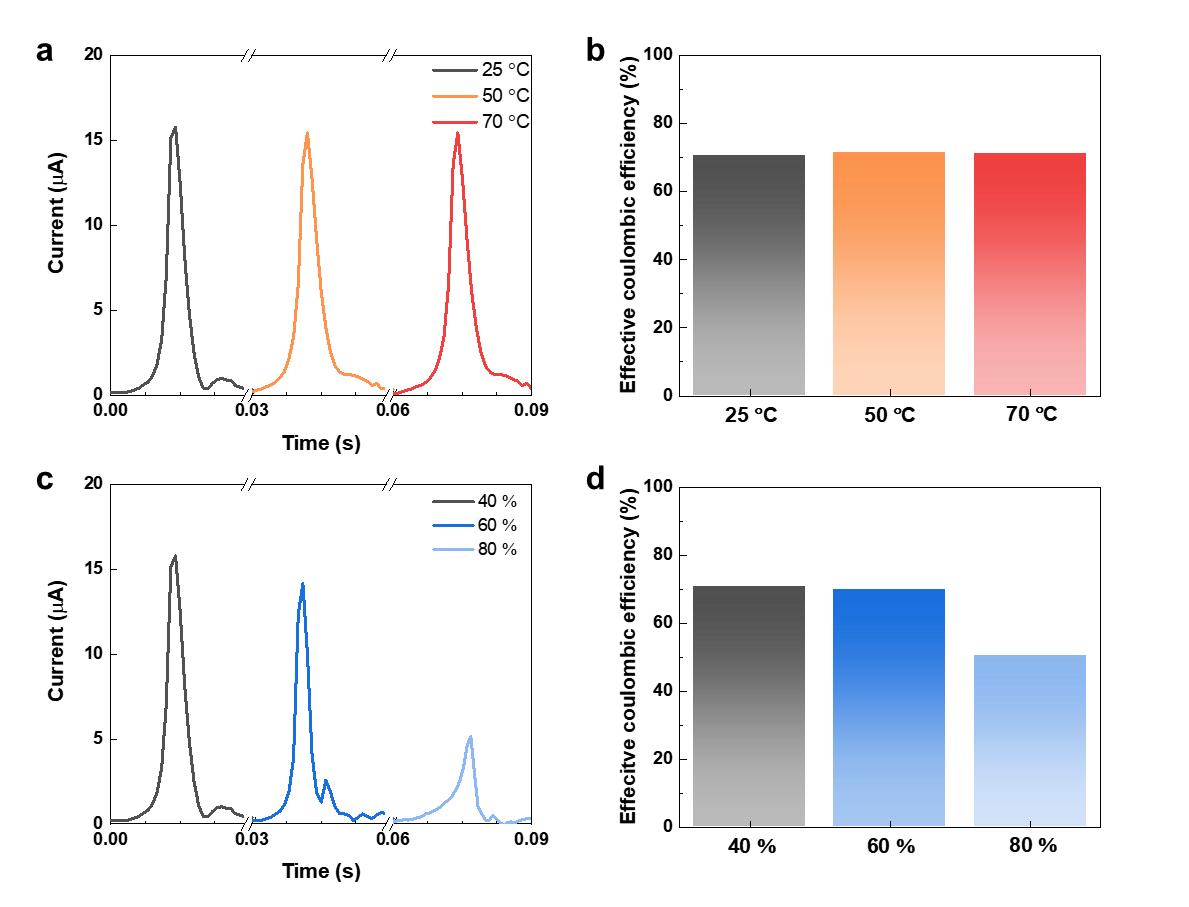
**

**Fig. S15** (**a**) Effect of temperature on output current of TENGs and (**b**) corresponding effective Coulombic efficiency of TENG−SCs. (**c**) Effect of humidity on output current and (**d**) corresponding effective Coulombic efficiency of TENG−SCs


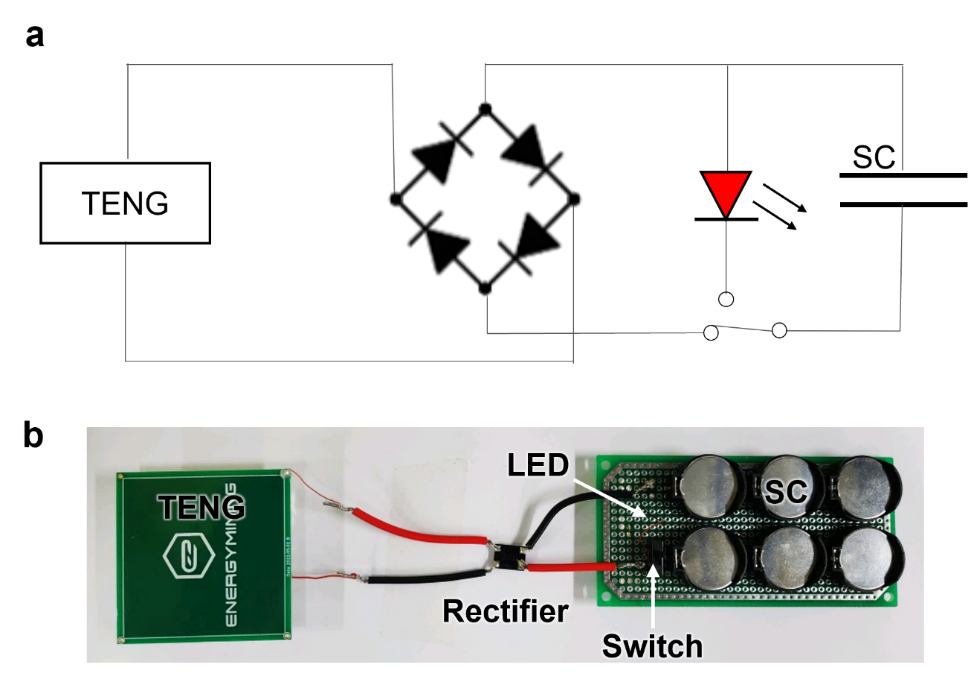


**Fig. S16** (**a**) Circuit diagram and (**b**) photographs showing the test circuit used for the TENG–SC hybrid device to illuminate an LED


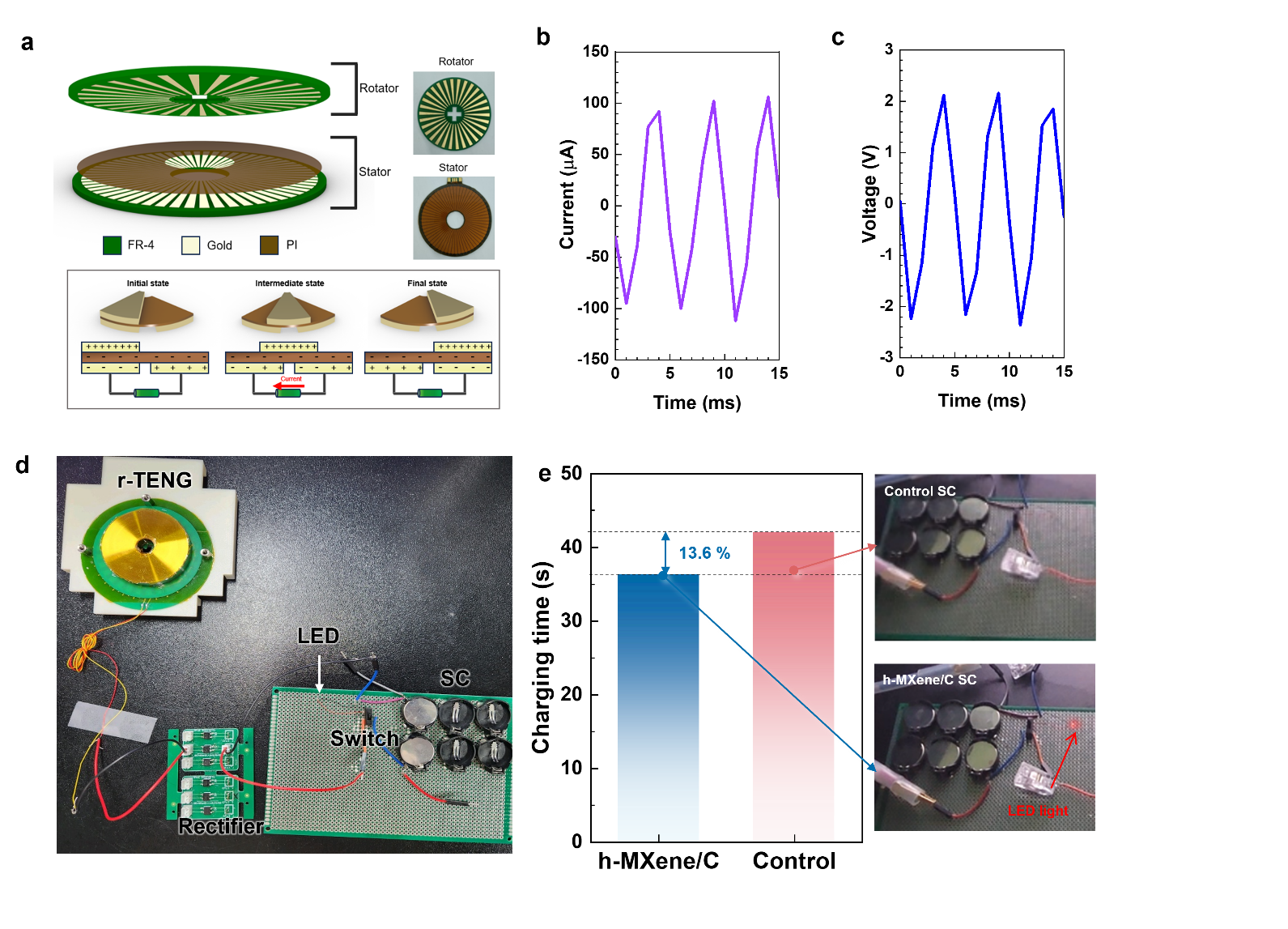


**Fig. S17 a** Schematic representation of the r-TENG components and its energy harvesting mechanism. (**b**) Output current and (**c**) voltage of the r-TENG. **d** Photograph of the test circuit for the h-MXene/C SC−rTENG hybrid device. **e** Comparison of the charging time required for the r-TENG–SC hybrid devices (h-MXene/C SC vs. control SC) to power an LED, with photographs on the right showing the devices after 36 s of charging


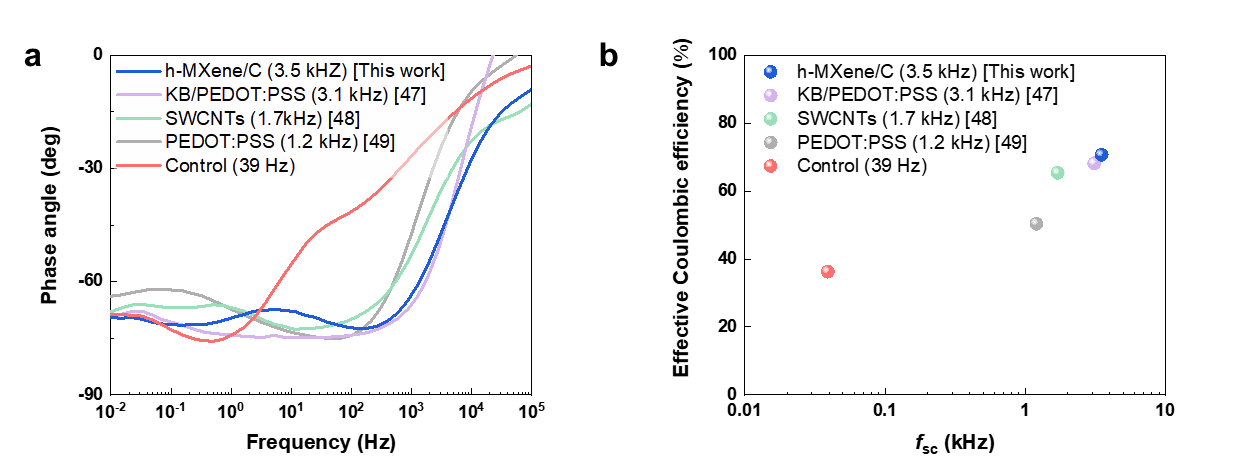


**Fig. S18** Comparison in (**a**) bode plots of the h-MXene/C and previously reported high-frequency SCs and (**b**) corresponding effective Coulombic efficiency (η) of TENG–SC hybrid devices as a function of *f*_SC_ [47-49]. The values in parentheses in the legend represent their *f*_SC_


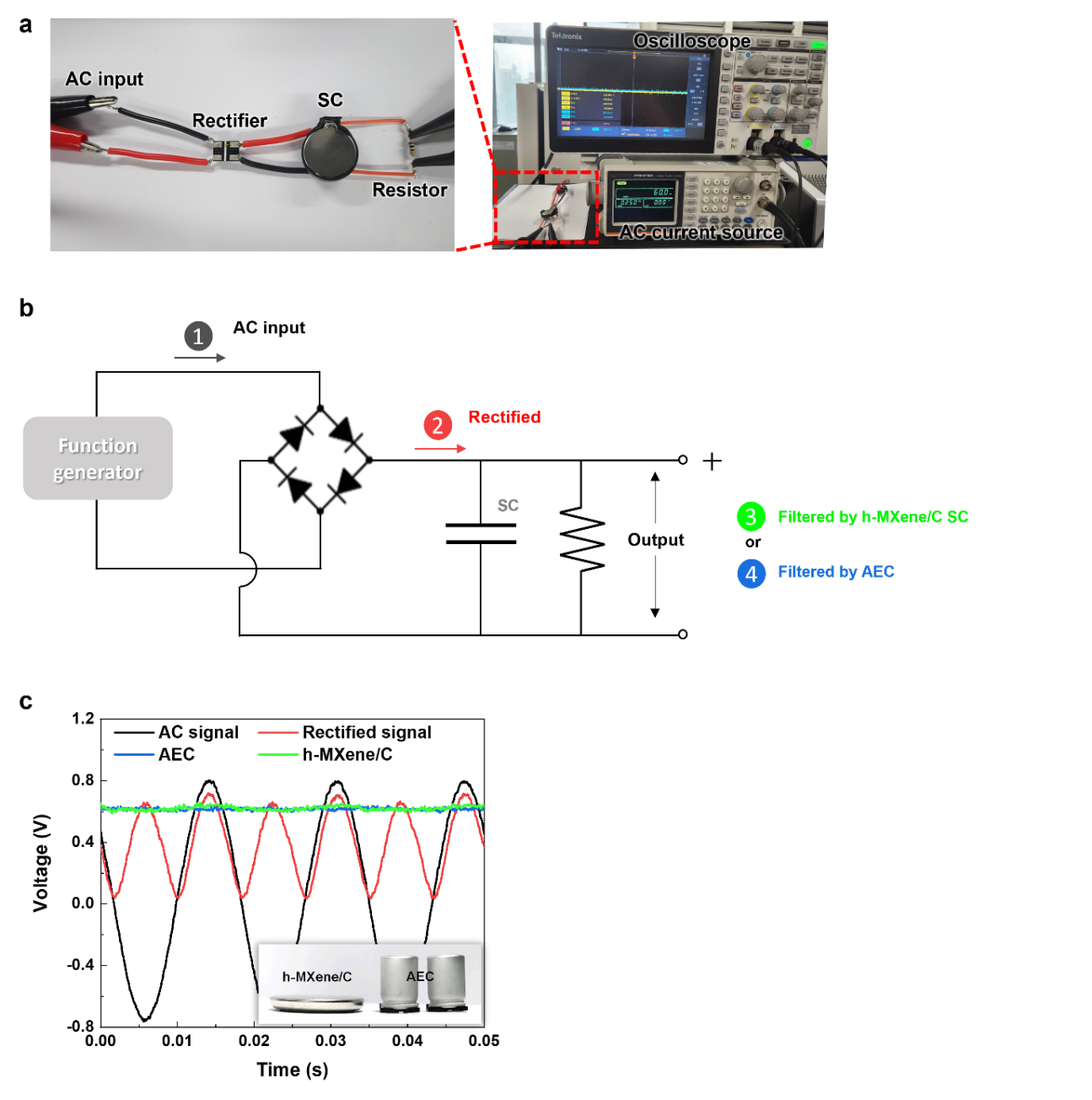


**Fig. S19** (**a**) Photographs and (**b**) circuit diagram depicting the experimental setup for testing the AC line-filtering capability of the h-MXene/C SC. (**c)** Voltage profiles illustrating the AC line-filtering performance of the h-MXene/C SC, with an inset showing the size reduction advantage of the h-MXene/C SC compared to a conventional AEC of equivalent capacitance


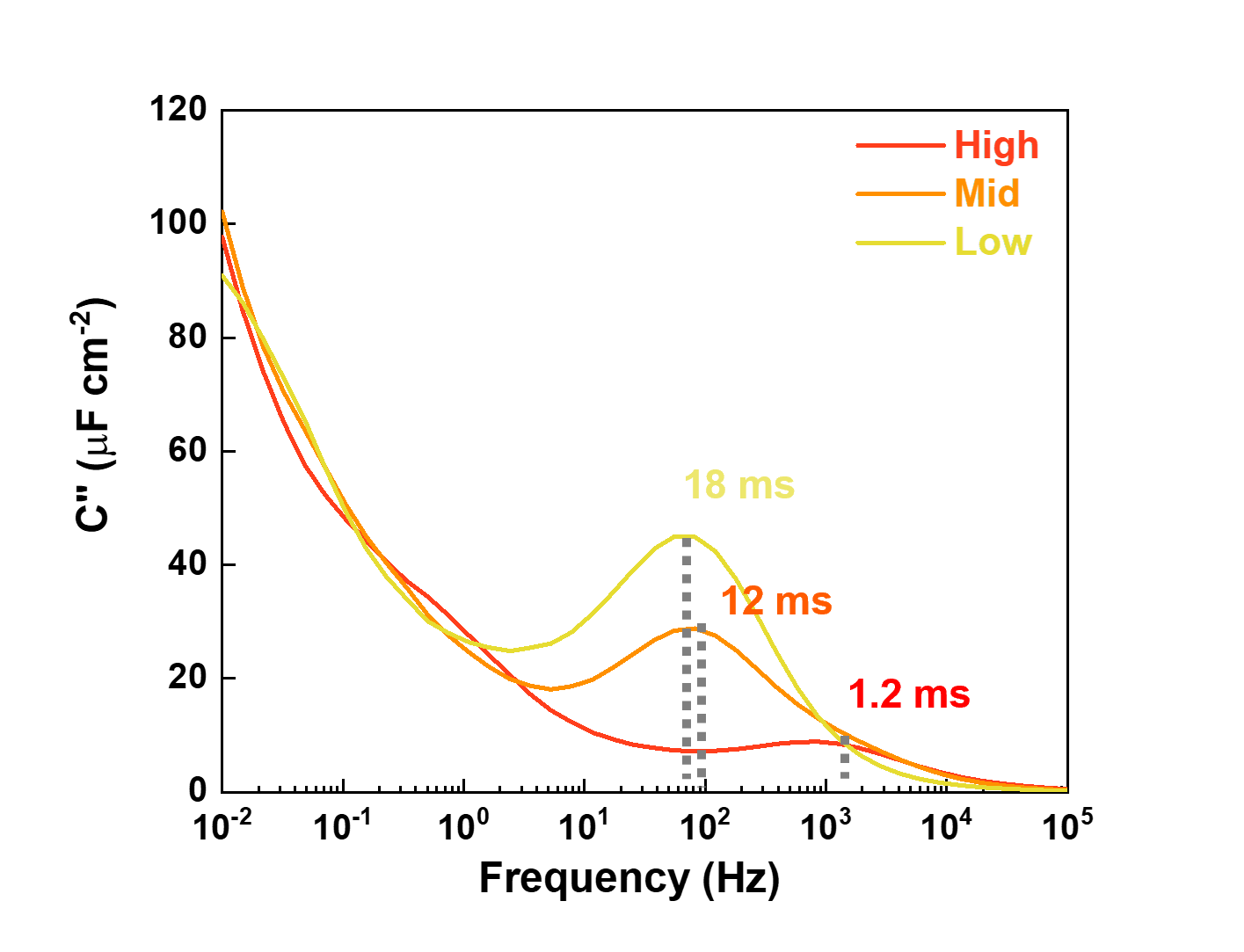


**Fig. S20** Frequency-dependent areal capacitance of the model SCs

**
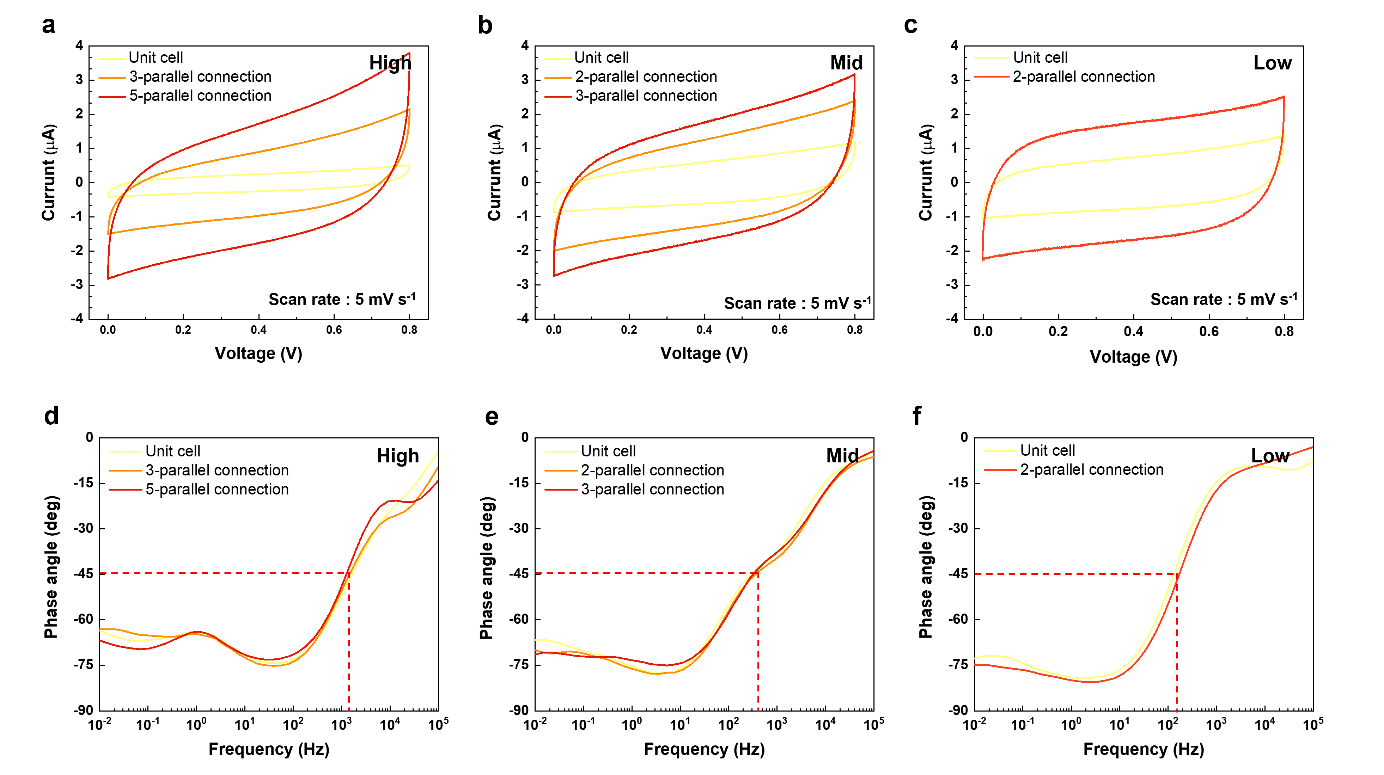
**

**Fig. S21** CV profiles (scan rate = 5 mV s^−1^) and Bode plots for three sets of model SCs: (**a** and **d**) High-SCs, (**b** and **e**) Mid-SCs, and (**c** and **f**) Low-SCs

**
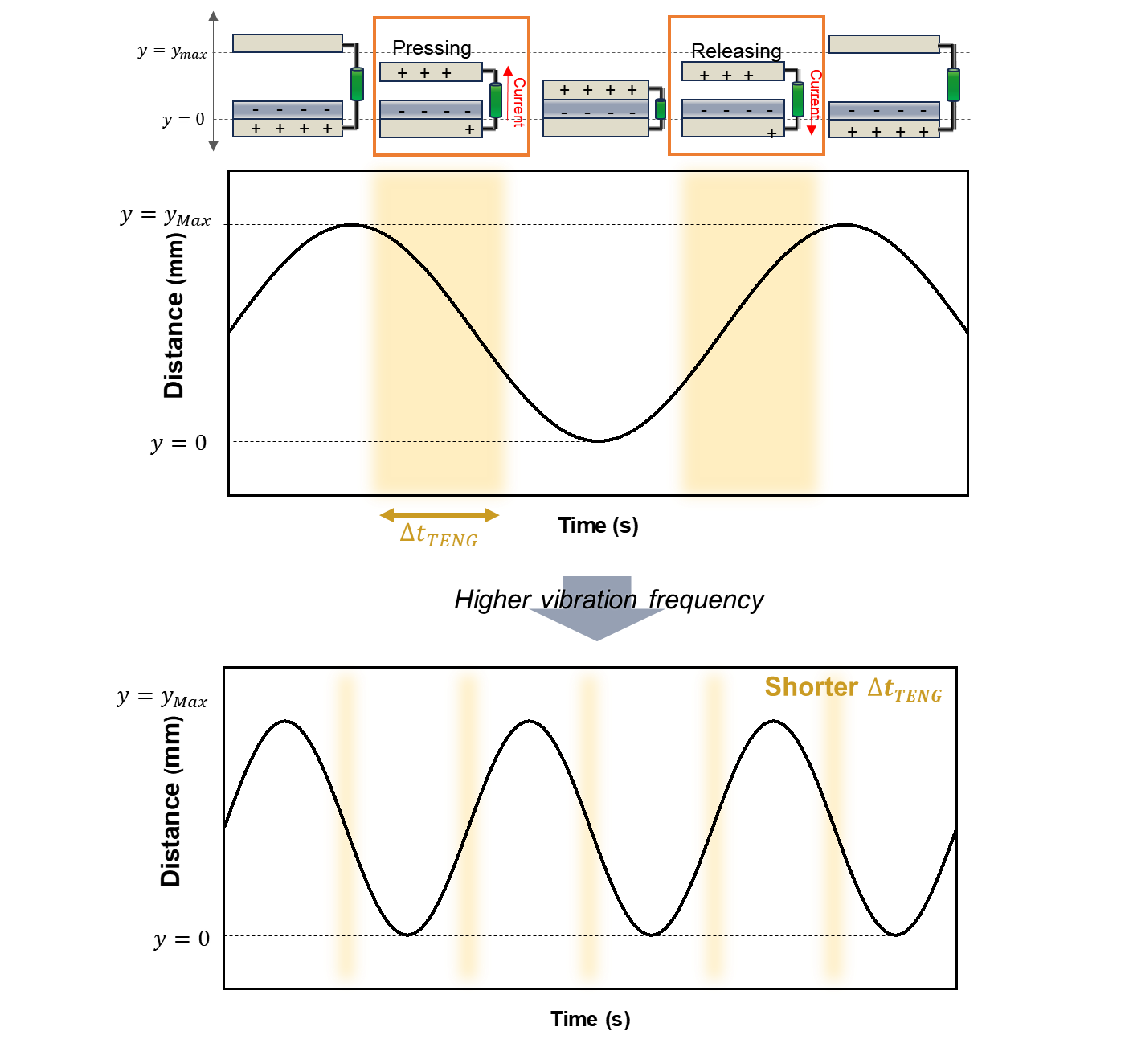
**

**Fig. S22** Schematic representation of the effect of vibration frequency on the Δt_TENG_

**
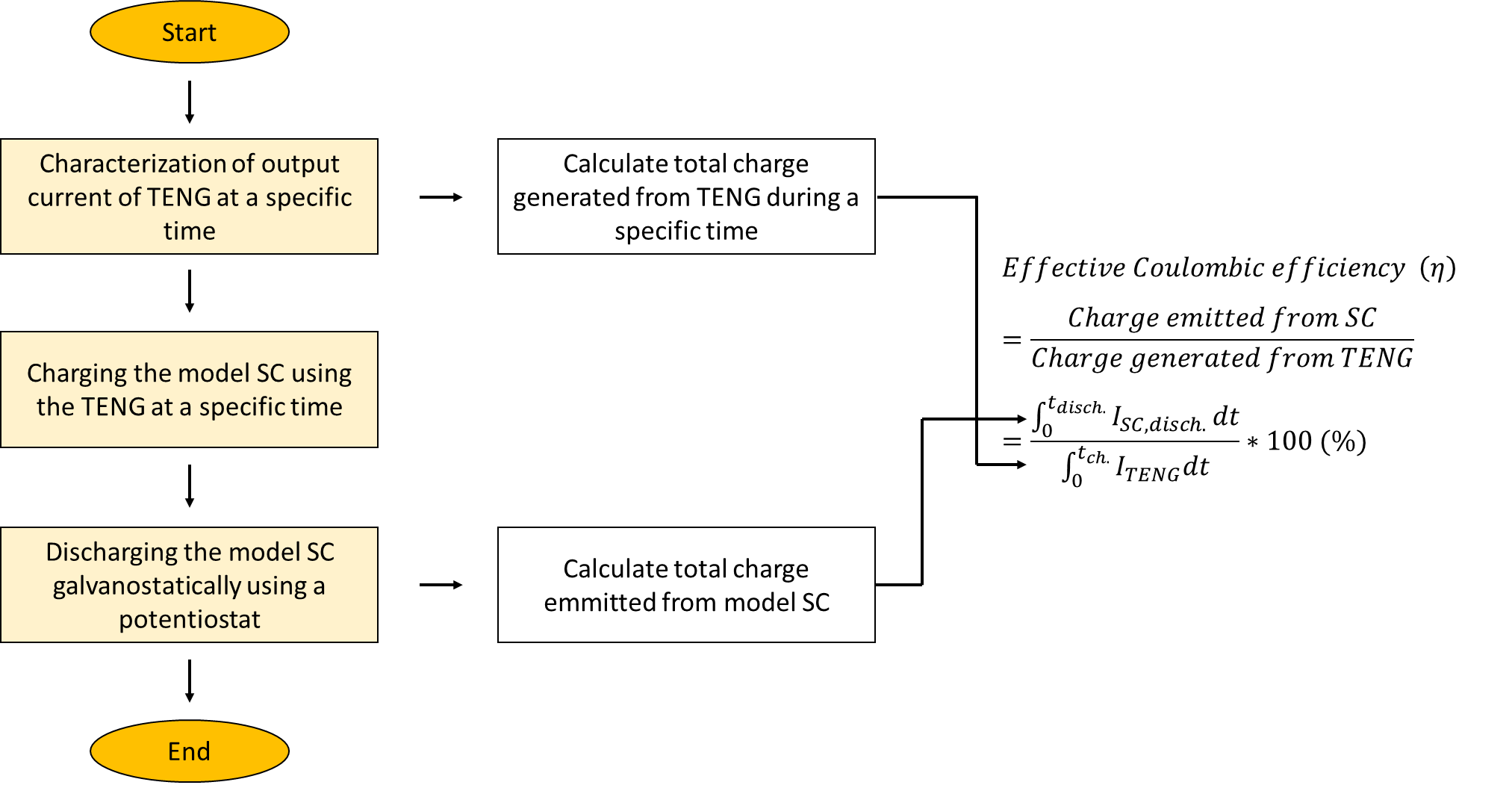
**

**Fig. S23** An experimental flowchart illustrating the steps for calculating the effective Coulombic efficiency

**
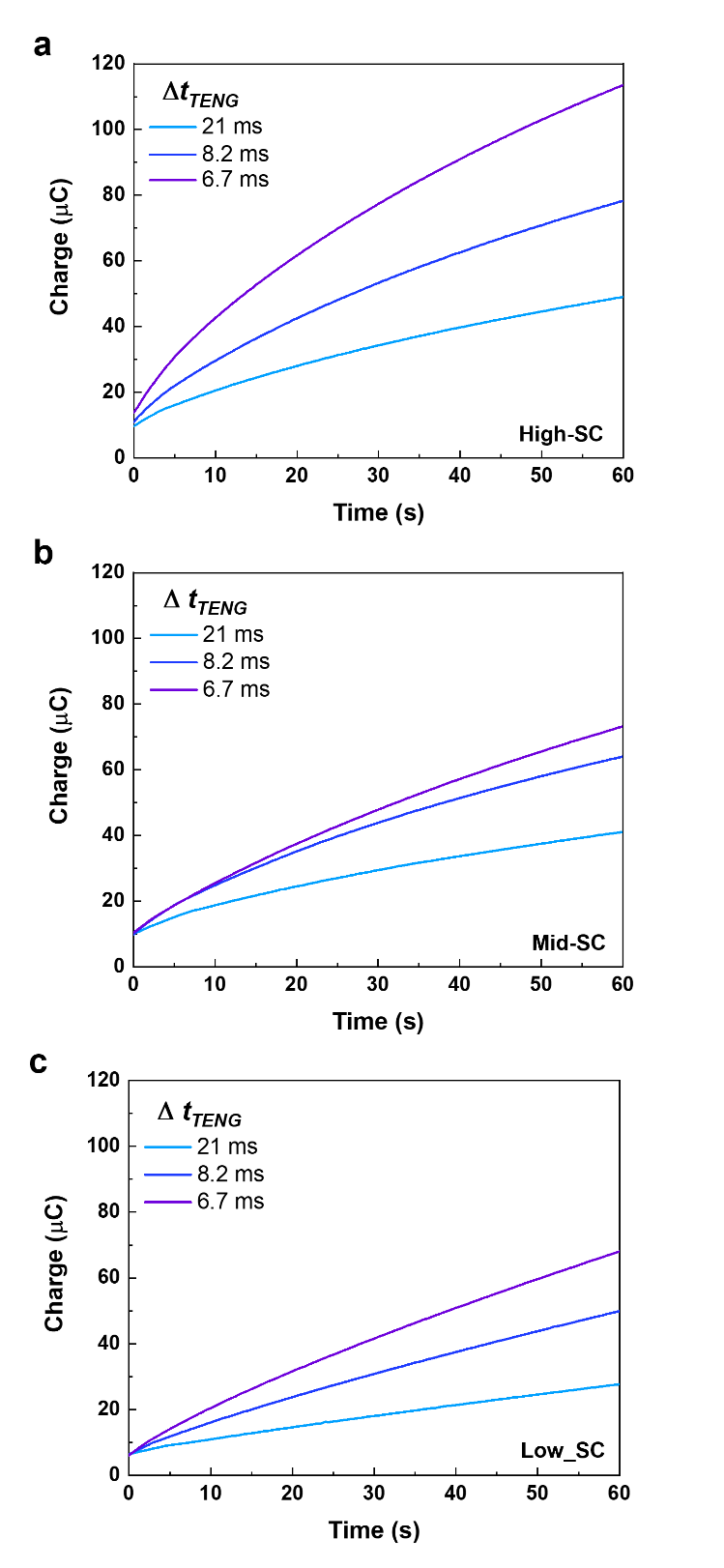

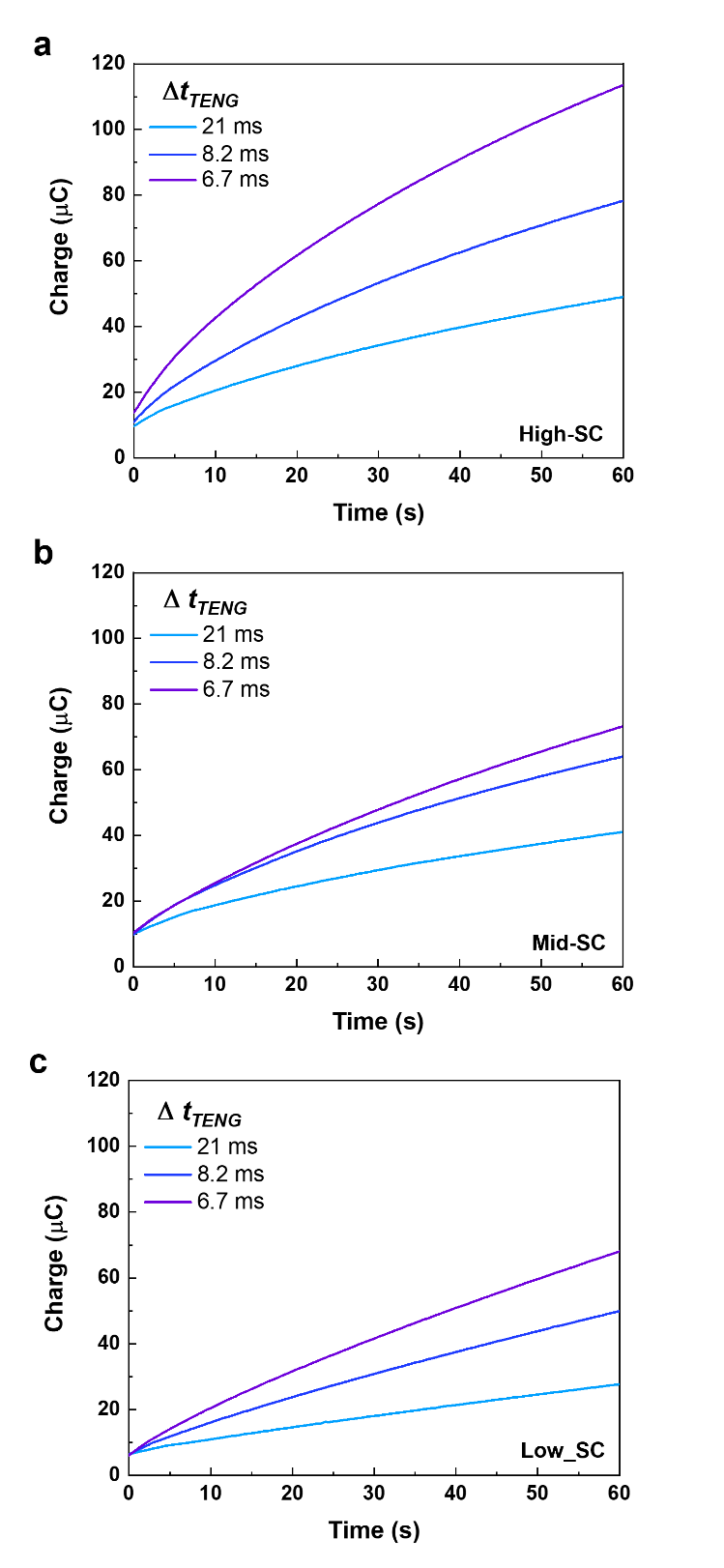
**

**
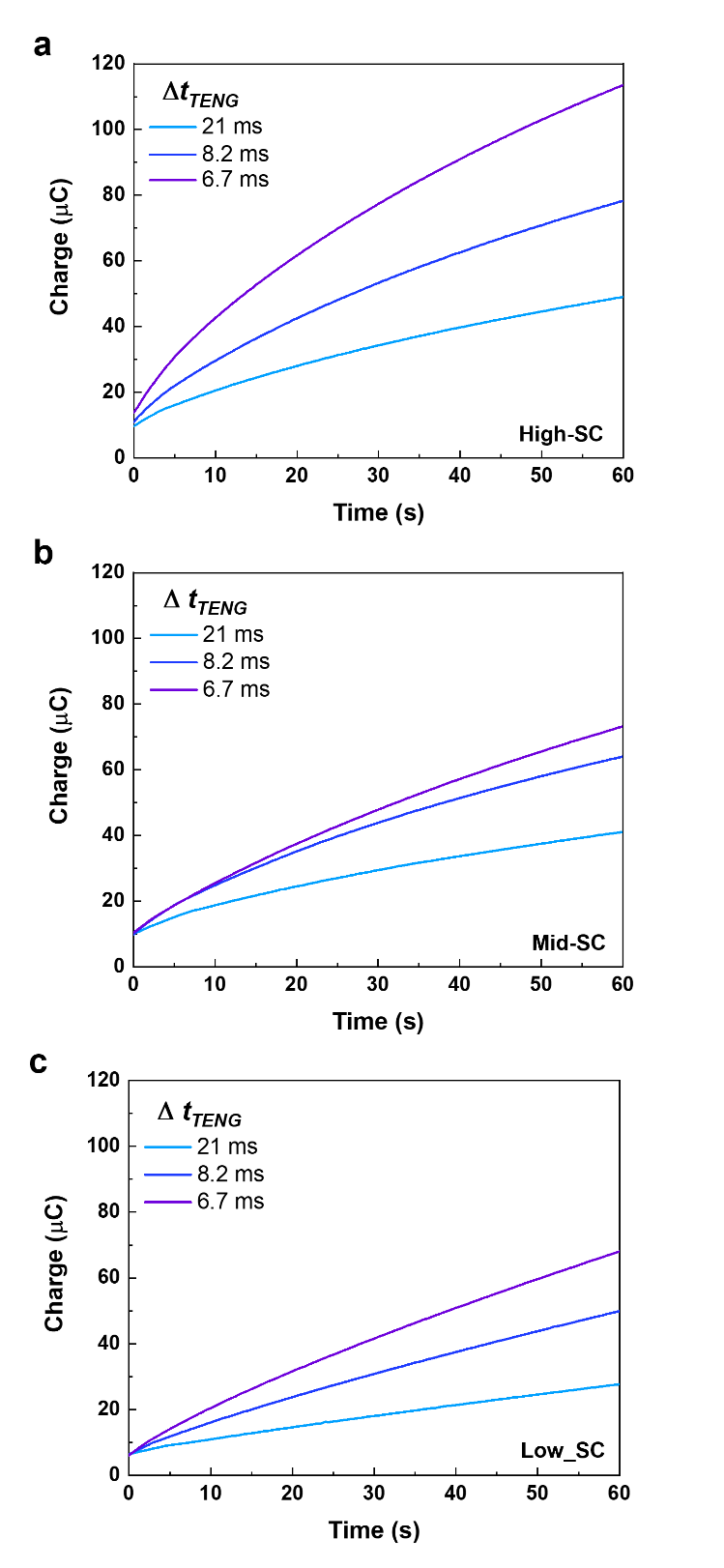
**

**Fig. S24** Real-time charge profiles stored in the (**a**) High-SCs, (**b**) Mid-SCs, and (**c**) Low-SCs during TENG charging with varying Δt_TENG_


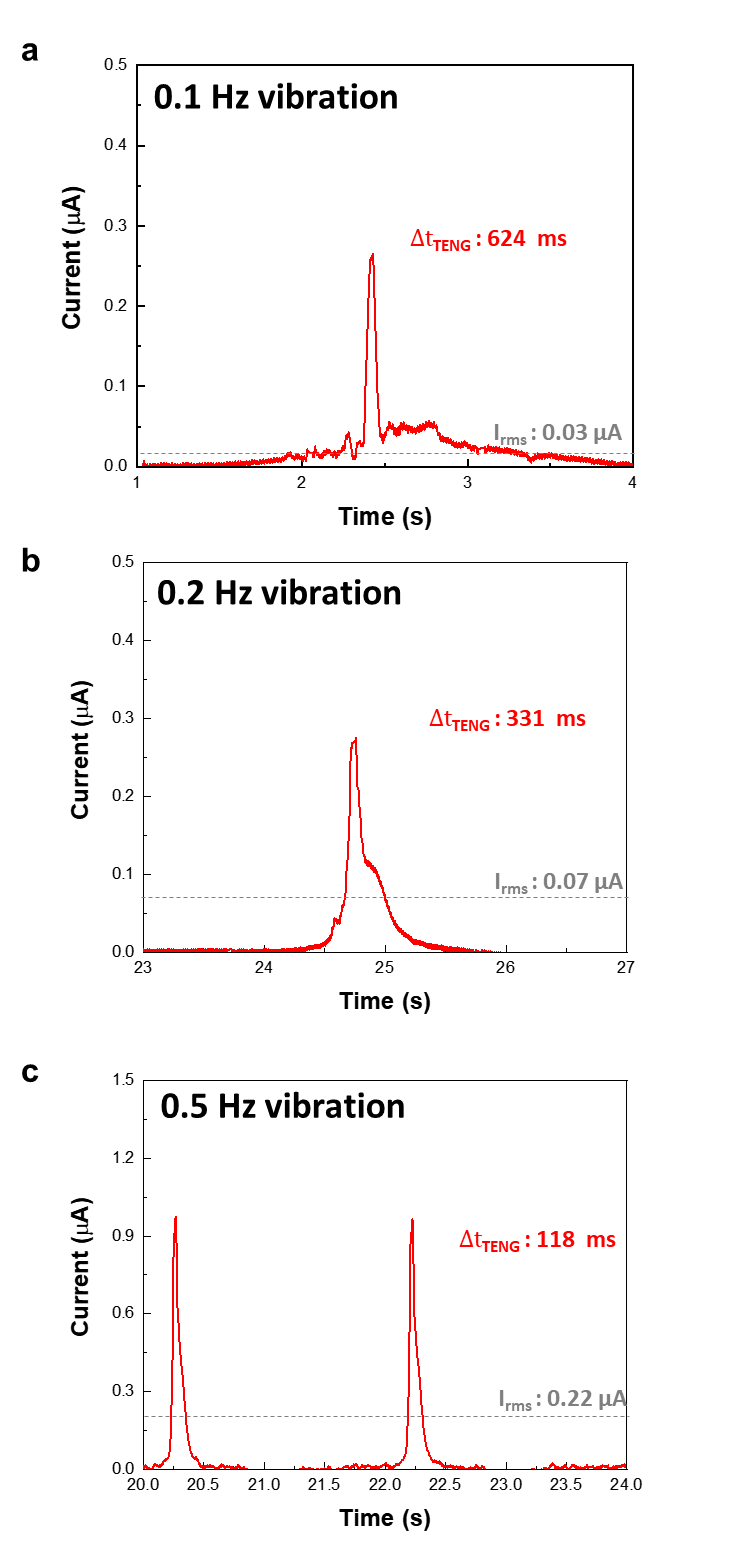

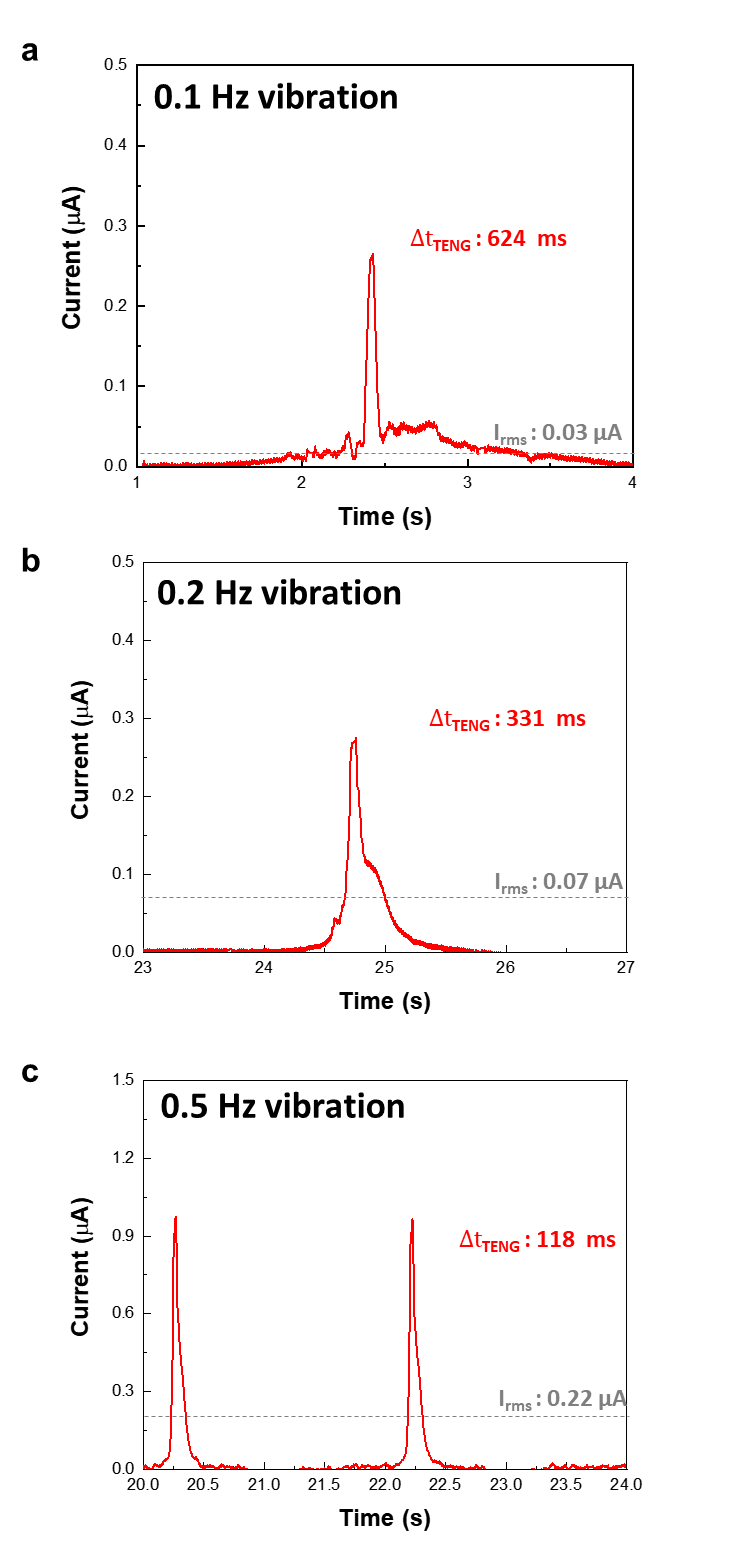

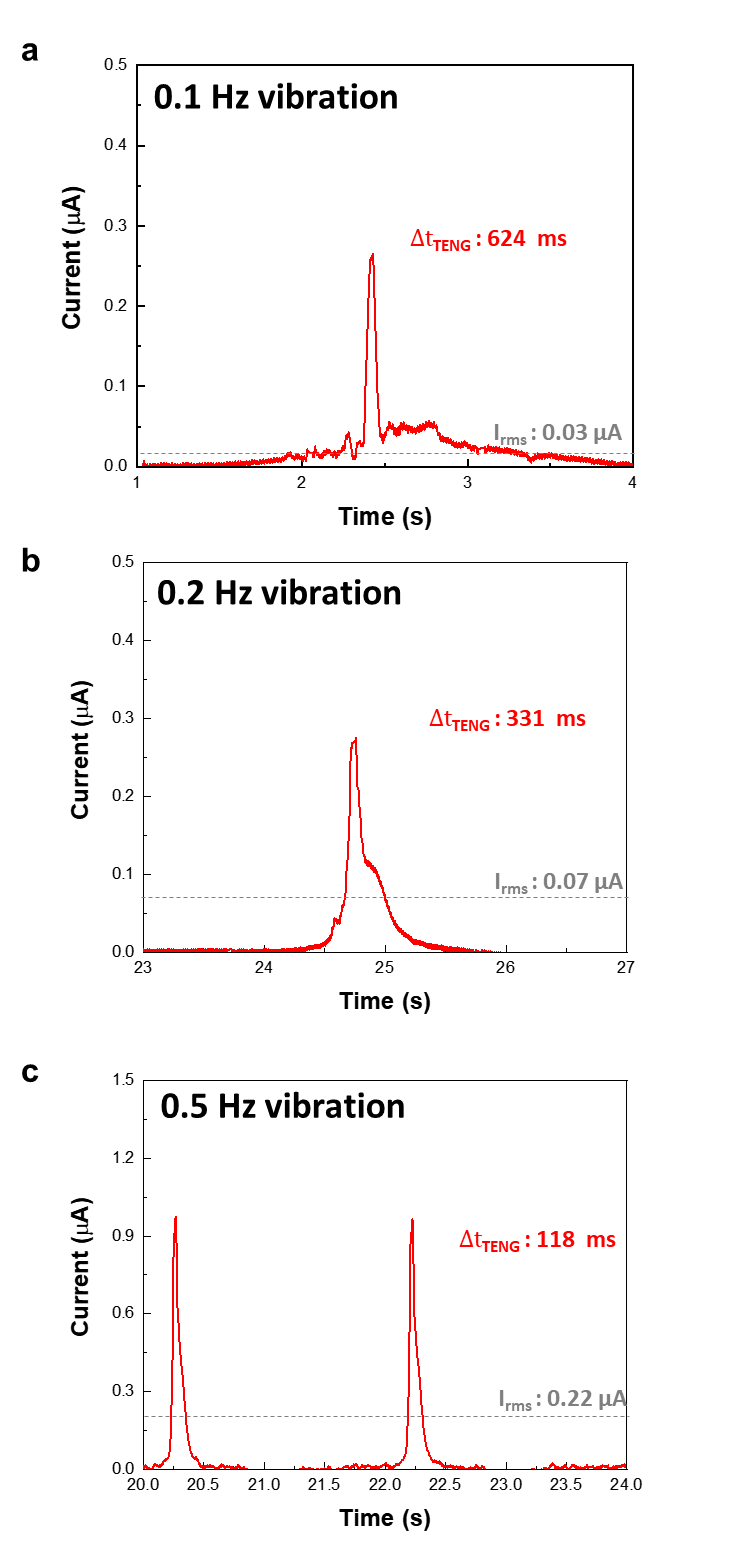


**Fig. S25** Output current of TENG at extremely low vibration frequencies. (a) 0.1Hz, (b) 0.2 Hz, and (c) 0.5 Hz)**Supplementary Tables**

**Table S1** Fabrication details and electrochemical properties of electrode components for SCs used in the Fig. S18 [47-49].

|  | **Fabrication** | **Active  material** | **Electrode thickness  (μm)** | **Electrolyte** | **Characteristic frequency (kHz)** | **References** |
| --- | --- | --- | --- | --- | --- | --- |
| **SWCNT** | Vacuum filtration | SWCNT | 0.3 | EMIM-TFSI | 1.7 | [47] |
| **PEDOT:PSS** | Spin coating | PEDOT:PSS | 0.3 | EMIM-TFSI | 1.2 | [48] |
| **KB/PEDOT:PSS** | Vacuum filtration | Ketjenblack/ PEDOT:PSS | 0.2 | EMIM-TFSI | 3.1 | [49] |

**Table S2** Fabrication details and properties of electrode components for the three model SCs

|  | **Fabrication method** | **Porosity**  **[%]** | **Electronic conductivity**  **[S cm^-1^]** | **Active material** | **Binder** | **Composition (Active material/ binder = w/w)** | **Thickness [μm]** |
| --- | --- | --- | --- | --- | --- | --- | --- |
| **High** | Spray | 58.3 | 73.31 | Super P | PEDOT:PSS | 2/1 | 1.2 |
| **Mid** | Spray | 44.2 | 68.33 | Super P | PEDOT:PSS | 2/1 | 5.2 |
| **Low** | Slurry-casting | 18.6 | 4.723 | Super P | CMC | 7/3 | 15.3 |

**Table S3** Configuration and total capacitance of the three model SCs connected in parallel

|  | **Capacitance of unit cell [μF]** | **# of parallel connected cells** | **Total capacitance [μF]** |
| --- | --- | --- | --- |
| **High** | 57 | 5 | 322 |
| **Mid** | 108 | 3 | 312 |
| **Low** | 147 | 2 | 311 |

**Supplementary References**

1. Z. Fan, N. Islam, S. B. Bayne. Towards kilohertz electrochemical capacitors for filtering and pulse energy harvesting. Nano Energy **39**, 306-320 (2017). <https://doi.org/10.1016/j.nanoen.2017.06.048>
2. J. R. Miller, R. A. Outlaw, B. C. Holloway. Graphene double-layer capacitor with ac line-filtering performance. Science **329**, 1637 (2010). <https://doi.org/10.1126/science.1194372>
3. K. Sheng, Y. Sun, C. Li, W. Yuan, G. Shi. Ultrahigh-rate supercapacitors based on eletrochemically reduced graphene oxide for ac line-filtering. Sci. Rep. **2**, 247 (2012). <https://doi.org/10.1038/srep00247>
4. M. Wu, F. Chi, H. Geng, H. Ma, M. Zhang et al. Arbitrary waveform AC line filtering applicable to hundreds of volts based on aqueous electrochemical capacitors. Nat. Commun. **10**, 2855 (2019). <https://doi.org/10.1038/s41467-019-10886-7>
5. Q. Jiang, N. Kurra, K. Maleski, Y. Lei, H. Liang et al. On‐chip MXene microsupercapacitors for ac‐line filtering applications. Adv. Energy Mater. **9**, 1901061 (2019). <https://doi.org/10.1002/aenm.201901061>
6. H. Tang, Y. Tian, Z. Wu, Y. Zeng, Y. Wang et al. AC line filter electrochemical capacitors: materials, morphology, and configuration. Energy Environ. Mater. **5**, 1060-1083 (2022). <https://doi.org/10.1002/eem2.12285>
7. N. Kurra, M. K. Hota, H. N. Alshareef. Conducting polymer micro-supercapacitors for flexible energy storage and Ac line-filtering. Nano Energy **13**, 500-508 (2015). <https://doi.org/10.1016/j.nanoen.2015.03.018>
8. G. S. Gund, J. H. Park, R. Harpalsinh, M. Kota, J. H. Shin et al. MXene/polymer hybrid materials for flexible AC-filtering electrochemical capacitors. Joule **3**, 164-176 (2019). <https://doi.org/10.1016/j.joule.2018.10.017>
9. J. Luo, Z. L. Wang. Recent advances in triboelectric nanogenerator based self-charging power systems. Energy Storage Materials **23**, 617-628 (2019). <https://doi.org/10.1016/j.ensm.2019.03.009>
